# Derivatives of the triaminoguanidinium ion, 5. Acylation of triaminoguanidines leading to symmetrical tris(acylamino)guanidines and mesoionic 1,2,4-triazolium-3-aminides

**DOI:** 10.3762/bjoc.13.57

**Published:** 2017-03-22

**Authors:** Jan Szabo, Julian Greiner, Gerhard Maas

**Affiliations:** 1 Institute of Organic Chemistry I, Ulm University, Albert-Einstein-Allee 11, D-89081 Ulm, Germany

**Keywords:** guanidines, mesoionic compounds, triaminoguanidinium salts, 1,2,4-triazolium-3-aminides

## Abstract

Depending on the reaction conditions, *N*,*N*’,*N*’’-tris(benzylamino)guanidinium salts can react with carboxylic acid chlorides to form either symmetrical *N*,*N*’,*N*’’-tris(*N*-acyl-*N*-benzylamido)guanidines **6** or mesoionic 4-amino-1,2,4-triazolium-3-hydrazinides **7**. The latter were converted into 1,2,4-triazolium salts by protonation or methylation at the hydrazinide nitrogen atom. Neutral 1,2,4-triazoles **10** were obtained by catalytic hydrogenation of an *N*-benzyl derivative. Crystal structure analyses of a 4-benzylamino-1,2,4-triazolium-3-hydrazinide and of two derived 1,2,4-triazolium salts are presented.

## Introduction

Easily accessible by the reaction of guanidinium chloride with hydrazine [2], triaminoguanidinium chloride (TAG-Cl) offers the opportunity to serve as a *C*_3_-symmetrical platform for the synthesis of triaminoguanidines with multiple functionalization (see [3] and references cited there). In most studies reported so far, triaminoguanidinium salts or the neutral triaminoguanidine have been reacted with carbonyl compounds. Reactions with aldehydes [3–7] or ketones [8–10] yielded the corresponding tris(iminyl)guanidines; cyclocondensation of pentane-2,4-dione at one hydrazinyl branch of TAG-Cl afforded a pyrazole which went on to a 3,6-di(pyrazol-1-yl)-1,2,4,5-tetrazine [11], and with 1,1,1-trifluoro-2,4-pentanedione two branches of TAG-Cl were converted into pyrazoline moieties and the third one into an enaminone [12].

**Scheme 1:**
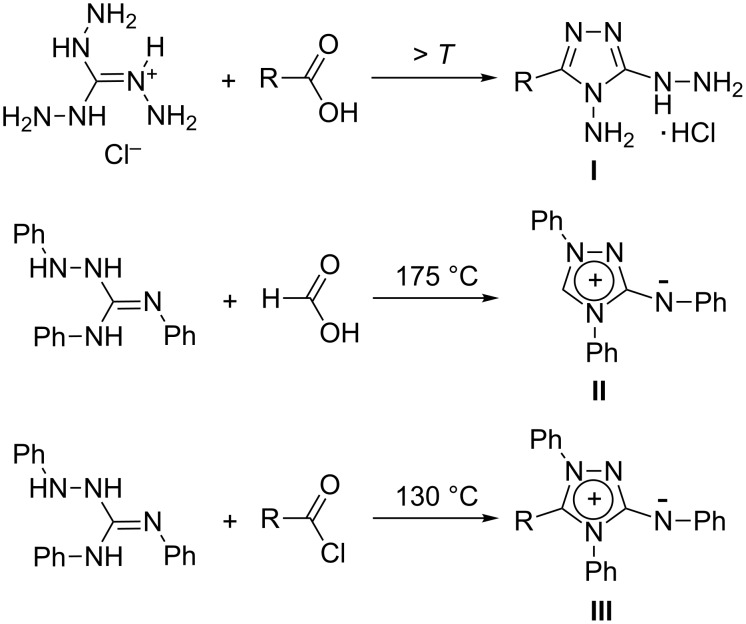
Reactions of aminoguanidines with carboxylic acids and acid chlorides. The structural formulae shown for **II** and **III**, suggesting a positively charged heteroaromatic ring system, are discontinued nowadays, because they do not represent the bond structure appropriately (vide infra).

Not much is known about the acylation of triaminoguanidines. Reactions with carboxylic acids have been addressed only rarely, reactions with acid chlorides appear to be unknown. We have recently reported on the threefold carbamoylation of *N*,*N*’,*N*’’-tris(benzylamino)guanidinium salts with aryl isocyanates [3]. Concerning the reaction with carboxylic acids, it is known that TAG-Cl and formic acid on heating yield 3-hydrazinyl-4-amino-4*H*-1,2,4-triazole hydrochloride (**I**, R = H) (Scheme 1) [13]. With the higher homologs of formic acid, the authors of that study observed the formation of resinous materials only. In a recent paper, however, evidence for the formation of the corresponding derivatives of **I** (R = Me, CF_3_, Ph, ClCH_2_) in good yields was presented, although they were transformed further without isolation [14]. An old observation with a long-lasting impact was made by M. Busch who studied the reaction of formic acid with triphenylaminoguanidine: the originally assumed bicyclic constitution of the reaction product, called “nitron” [15–16], was much later recognized [17] and structurally confirmed [18] as the mesoionic 1,2,4-triazolium-3-aminide **II** (Scheme 1). “Nitron” became known as an analytical reagent for the quantitative determination of nitrate [16,19], perchlorate, and some metals [20].

In subsequent studies, Busch and co-workers found that a variety of 5-substituted 1,2,4-triazolium-3-aminides **III** (Scheme 1) can be prepared either in one step from triphenylaminoguanidine and acid chlorides at elevated temperatures or in two steps using an aldehyde and subsequent oxidation [21–22]. Several decades later, a range of diversely substituted 1,2,4-triazolium-3-aminides were prepared by condensation of *N*-amino-*N*-R-*N*’-phenylbenzamidines with phenylisocyanide dichloride [23] or 4-aryl-1,1-dibromo-2,3-diazabutadienes [24].

The 1,2,4-triazolium-3-aminides **II** and **III** belong to the family of mesoionic five-membered heterocycles, of which the sydnones (1,2,3-oxadiazolium-5-olates), sydnone imines (1,2,3-oxadiazolium-5-aminides), and 1,3,4-thiadiazolium derived mesoionics are perhaps the best known members [25–26]. While not much is known about congeners of **II**, diverse biological activities have been reported for some of the other types of mesoionic heterocycles. For example, antitumor [27–29], antileishmanial [30] and trypanocidal [31] activities, as well as reduction of the phosphorylation efficiency of rat liver mitochondria [32], have been described for 1,3,4-thiadiazolium-2-phenylaminides.

We present now the results of our studies on the acylation of triaminoguanidines with carboxylic acid chlorides. Further, we show that *N*,*N*’,*N*’’-tris(benzylamino)guanidine reacts with acid chlorides to afford either the threefold *N*-acylation product or a mesoionic 1,2,4-triazolium-3-aminide, depending on the reaction conditions. Because of the long-standing interest in the bond structure of mesoionic compounds, we have also conducted some structural and spectroscopic studies of the new 1,2,4-triazolium-3-aminides and the 3-amino-1,2,4-triazolium salts derived thereof [33].

## Results and Discussion

### Acylation of triaminoguanidine

**Scheme 2:**
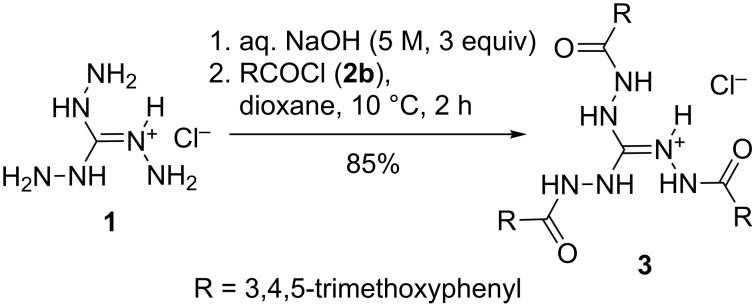
Threefold *N*-acylation of triaminoguanidinium chloride (**1**) with acyl chloride **2b**.

Triaminoguanidine is highly soluble in water [34], and triaminoguanidinium chloride (TAG-Cl, **1**) is soluble in hot water or water/ethanol mixtures, but both are insoluble or sparingly soluble in common organic solvents. Since aqueous media cannot be avoided, these properties pose an obstacle to acylation reactions, which therefore are expected to succeed only with acylating reagents less sensitive to hydrolysis. We found indeed, that triaminoguanidine, generated in situ by deprotonation of **1** in strongly alkaline aqueous solution, could be acylated effectively with 3,4,5-trimethoxybenzoyl chloride (**2b**) to form the *N*,*N*’,*N*’’-tris(acylamino)guanidinium chloride **3** (Scheme 2). Hydrolysis of the acyl chloride is not a competitive reaction under these conditions, and formation of a 1,2,4-triazole, as observed for the reaction of **1** with formic acid at reflux conditions (see Scheme 1) also does not occur.

On the other hand, the analogous acylation reaction could not be performed with benzoyl chloride and acetyl chloride under various conditions, including the application of weaker bases (NEt_3_, Na_2_CO_3_) in dioxane and other aprotic-polar solvents.

### Reaction of 1,2,3-tris(benzylamino)guanidinium chloride with acyl chlorides

**Scheme 3:**
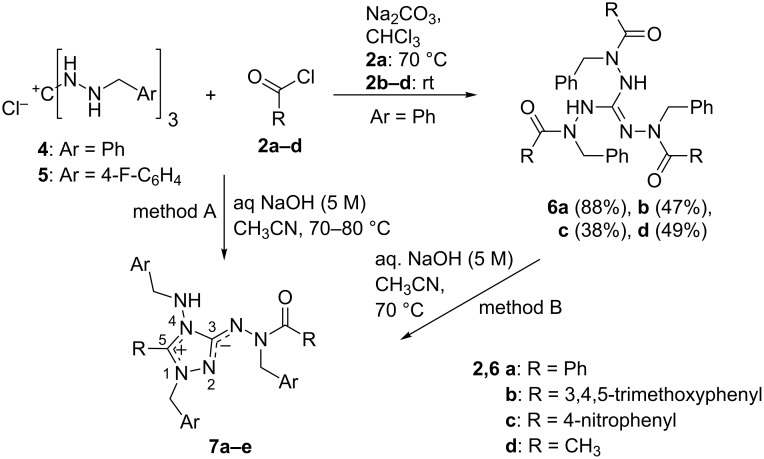
Reaction of 1,2,3-tris(benzylamino)guanidinium salts **4** and **5** with acyl chlorides to give 1,2,3-tris(acylamino)guanidines **6** and mesoionic 1,2,4-triazolium-3-aminides **7**. See Table 1 for Ar, R and yields of **7**.

As an example of *N*,*N*’,*N*’’-tris(alkylamino)guanidines, we have recently described the synthesis of *N*,*N*’,*N*’’-tris(benzylamino)guanidinium chloride (**4**) [3]. Since the direct alkylation of TAG-Cl (**1**) was not possible due to the solubility problem mentioned above, a two-step protocol – conversion of **1** into *N*,*N*’,*N*’’-tris(benzylideneamino)guanidinium chloride followed by catalytic hydrogenation of the imine groups – was developed. The fluorophenyl-substituted salt **5** was prepared analogously. Depending on the reaction conditions, the guanidinium salts **4** and **5** were found to react with acid chlorides **2** in two different ways (Scheme 3). When the acid chloride was added slowly to a solution of the guanidinium salt in chloroform in the presence of solid sodium carbonate, the expected *N*,*N*’,*N*’’-tris(acylamino)guanidines **6** were formed as the major products. Both the use of a weak base and a mild reaction temperature favor the formation of **6** at the expense of competitively formed mesoionic compounds **7**. This can be seen for the acylation of **4** with benzoyl chloride, where an elevated temperature was required for a sufficiently fast conversion and a mixture of **6a** and **7a** was obtained (Scheme 3 and Table 1).

**Table 1:** 1,2,4-Triazolium-3-aminides **7** synthesized (see Scheme 3).

Product	Ar	R	Method	Yield (%)

**7a**	Ph	Ph	A	47^a^
**7b**	Ph	3,4,5-(OMe)_3_-C_6_H_2_	A B	84 58
**7c**	Ph	4-NO_2_-C_6_H_4_	A^b^ B	41^b^ 50
**7d**	Ph	CH_3_	A B	48 70
**7e**	4-F-C_6_H_4_	CH_3_	A	75

^a^When the reaction **4** + **2a** was performed in CHCl_3_ at 70 °C with Na_2_CO_3_ as the base, **7a** could be isolated in ≈7% yield. ^b^K_2_CO_3_ instead of NaOH; **6c** (21%) was also obtained.

On the other hand, when the acylation reactions were conducted at elevated temperature in the presence of a stronger base (aqueous NaOH), mesoionic (1*H*-1,2,4-triazol-4-ium-3-yl)hydrazin-1-ides **7a–e** were formed in moderate to good yields (Scheme 3, method A; Table 1). With easily hydrolyzing 4-nitrobenzoyl chloride (**2c**), the alkaline medium had to be replaced by solid potassium carbonate, which resulted, however, in a separable mixture of **6c** and **7c**. It should be emphasized that, although the two procedures for the acylation of guanidinium salts **4** and **5** have been optimized to give either **6** or **7** selectively, the undesired product could be detected in some cases by characteristic signals in the ^1^H NMR spectra of the crude product mixtures.

It is reasonable to assume that the formation of betaines **7** according to method A (Scheme 3) proceeds via the neutral guanidine derivatives **6** as intermediates. In fact, the latter compounds are converted into betaines **7** under the same reaction conditions (method B). In terms of yields, however, the direct, one-step route (method A) turned out to be the better choice, as was found for betaines **7b–d** (Table 1).

All reactions shown in Scheme 3 were performed in the absence of air, since salts **4** and **5** suffer a partial oxidative degradation in the presence of oxygen under alkaline conditions. In fact, 3,4,5-trimethoxybenzamide was isolated in 20% yield when the reaction of **4** and **2b** (aq NaOH 5 M, CH_3_CN, 70 °C) was performed in the presence of air.

Similar to structurally related guanidines (*N*-ureido instead of *N*-acyl substitution [3]) and to *N*,*N*’,*N*’’-tris(isopropylideneamino)guanidine [10], the ^1^H NMR signals of tris(acylamino)guanidines **6** are in coalescence over a wide temperature range, without reaching the fast exchange regime up to 350 K. As an example, a ^1^H NMR spectrum of **6b**, recorded at 298 K, is shown in Figure S1 (Supporting Information File 1). This is a result of several dynamic processes, namely conformational and prototropic equilibria. The ^13^C NMR spectra of **6a–d**, on the other hand, indicate a static, unsymmetrical molecule around 300 K, showing for example separate signals for the three CH_2_ and C_carbonyl_ nuclei.

**Figure 1:**
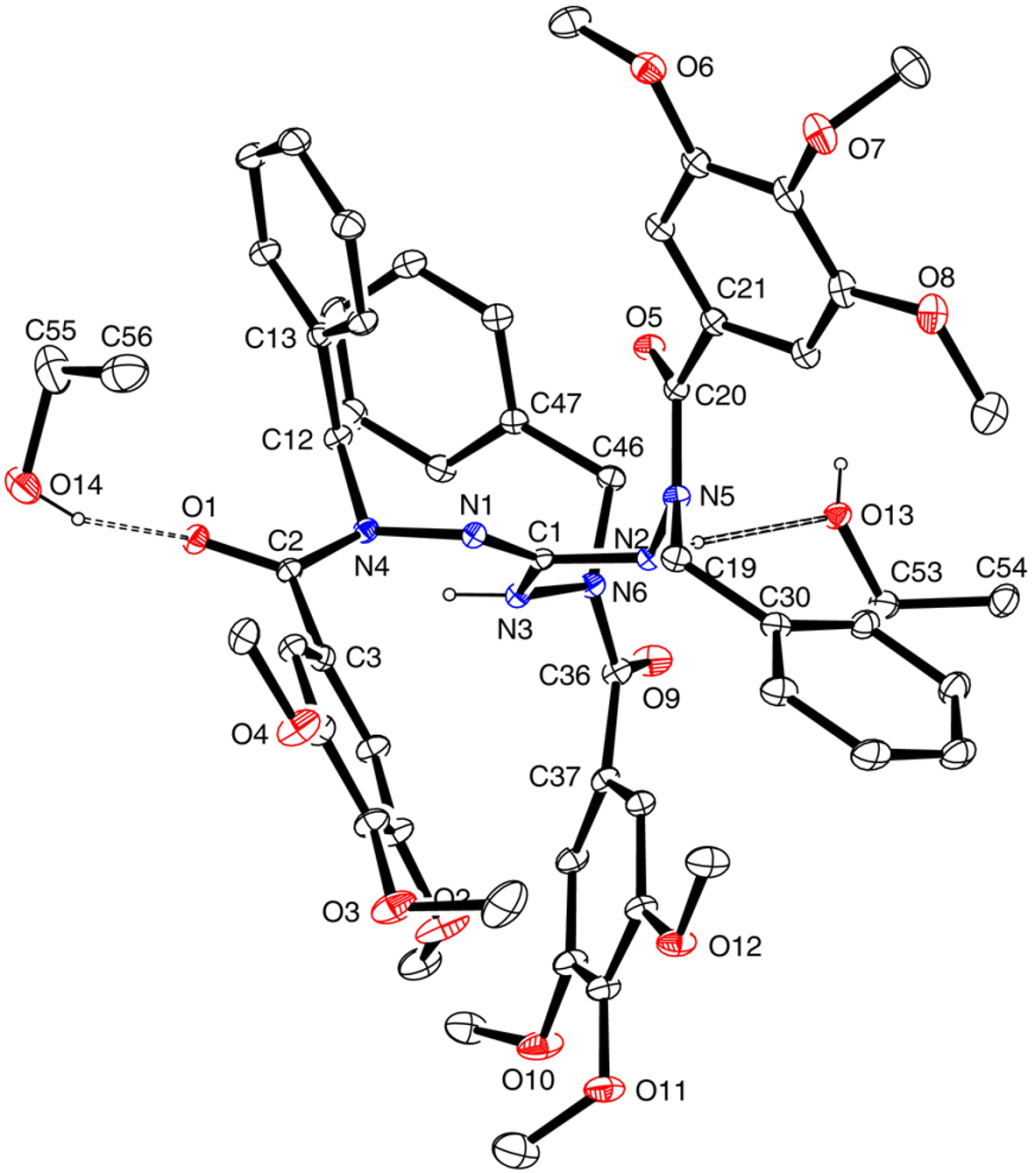
Molecular structure of **6b**·2C_2_H_5_OH in the solid state, with numbering of atoms (ORTEP plot). Selected bond lengths (Å): C1–N1 1.297(4), C1–C2 1.390(4), C1–N3 1.365(4). Selected bond angles (°): N1–C1–N2 117.3(3), N2–C1–N3 115.6(2), N1–C1–N3 126.9(3). Hydrogen bonds to the two ethanol molecules: N2–H···O13: N2···O13 3.060(1) Å, NH···O13 1.96(2) Å; N3–H···O14(1.5−*x, y*−0.*5*, 0.5−*z*): N3···O14 2.917(4) Å, NH···O14 2.11(2) Å; O13–H···O5(−*x*+1, -*y*, −*z*+1): O13···O5 2.733(3) Å, (O13–)H···O5 1.97 Å; O14–H···O1: O14···O1 2.846(4) Å, (O14–)H···O1 2.07(6) Å.

A single-crystal X-ray structure determination was performed for **6b**, which gave suitable crystals of **6b**·2C_2_H_5_OH when crystallized from ethanol. The guanidine structure could so be confirmed (Figure 1). It is interesting to note the unsymmetrical shape of the molecule: two benzyl groups are found above and one below the plane defined by the CN_3_ core of the guanidine. This is in contrast to the structure of the related 1,2,3-tris(1-benzyl-3-phenylureido)guanidine [3], where all three benzyl groups are placed on one side and the three polar ureido branches, interconnected by N–H···O hydrogen bonds, on the other side of the CN_3_ plane. In **6b**, on the other hand, the two guanidine N–H bonds and one of the carbonyl oxygen atoms are involved in hydrogen bonds to ethanol molecules.

**Scheme 4:**
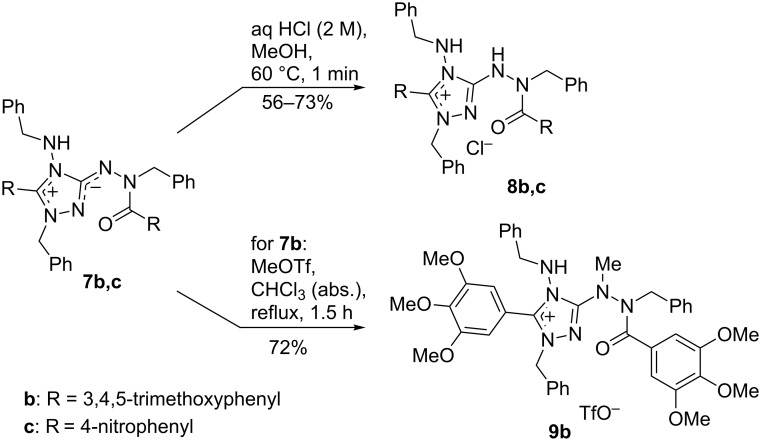
Protonation and methylation of 1,2,4-triazolium-3-aminides **7b**,**c**.

The mesoionic 1,2,4-triazolium-3-aminides **7a*–*e** are high-melting solids, the colors of which range from colorless (**7e**) to orange-red (**7c**). Their bond structure and characteristic spectroscopic data are discussed in the next section. A charge distribution as shown in the molecular formula (Scheme 3) is generally considered as being typical for these and related mesoionic compounds (see the next chapter). It was therefore expected that betaines **7** would be attacked by electrophiles at the exocyclic nitrogen atom bearing excess π-electron density. In fact the protonation of **7b**,**c** with hydrochloric acid gave rise to the 3-hydrazinyl-1,2,4-triazolium chlorides **8b**,**c** (Scheme 4) as well-crystallizing colorless or pale yellow solids. Analogously, the reaction of **7b** with methyl triflate afforded the 3-(1-methylhydrazinyl)-1,2,4-triazolium triflate **9b** by methylation of the exocyclic aminide nitrogen atom of the betaine; in contrast to other 1,2,4-triazolium-3-aminides [21,23], methylation with methyl iodide was not successful. Salt **9b** forms colorless hygroscopic crystals which assume an orange surface color when exposed to air for some minutes. The structures of 1,2,4-triazolium salts **8b** and **9b** were confirmed by single-crystal X-ray diffraction (vide infra).

**Scheme 5:**
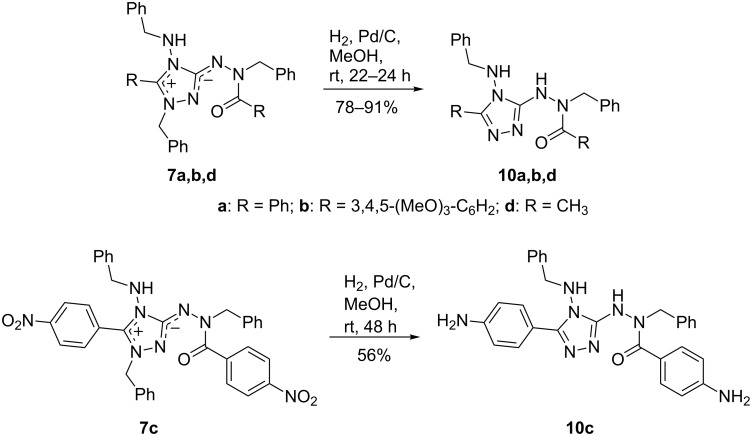
Catalytic hydrogenation/debenzylation of betaines **7**.

Catalytic hydrogenation of 1,2,4-triazolium-3-aminides **7** with H_2_ and Pd/C in methanol selectively cleaves the N1–C_benzyl_ bond and yields the neutral *N*-benzyl-*N*’-(4-benzylamino-4*H*-1,2,4-triazol-3-yl)benzohydrazides **10** in high yields (Scheme 5). In the case of **7c**, the nitro groups are concomitantly reduced to NH_2_ groups and the bis(aminophenyl) derivative **10c** is obtained. Thus, highly substituted and functionalized 1,2,4-triazoles can easily be prepared from TAG-Cl (**1**) in four steps.

### Solid-state structures of salts **8b** and **9b**

**Figure 2:**
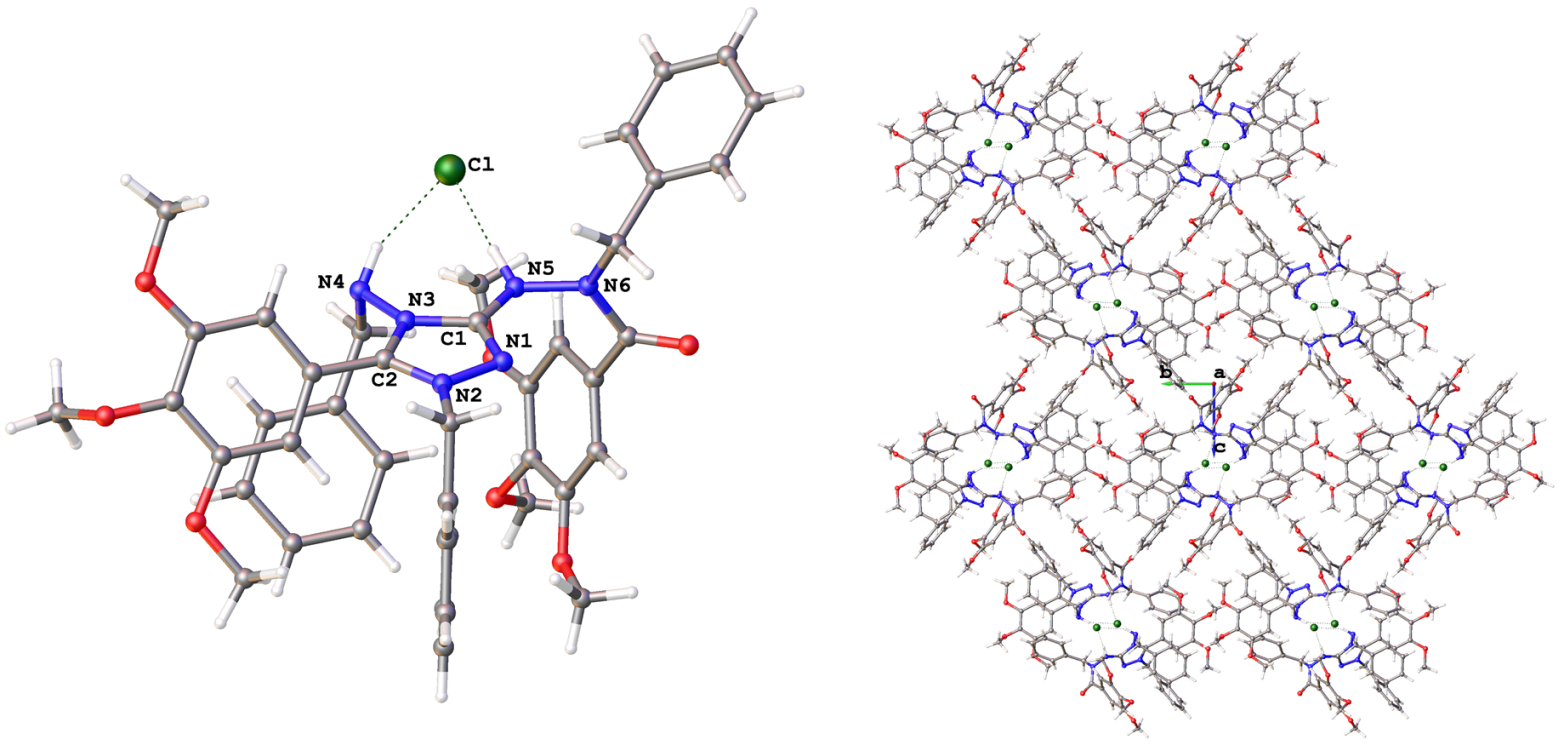
Left: molecular structure of **8b** in the solid state (OLEX2 plot). Right: crystal structure viewed along the crystallographic *a*-axis. Selected bond lengths (Å): C1–N1 1.317(2), C1–N3 1.372(2), C1–N5 1.349(2), N1–N2 1.388(2), C2–N2 1.319(2), C2–N3 1.352(2), N3–N4 1.412(2). Hydrogen bonds: N5–H···Cl: N5···Cl 3.060(1) Å, NH···Cl 2.11(2) Å, <(N5–H···Cl) 176(2)°; N4–H···Cl, N4···Cl 3.320(2) Å, NH···Cl 2.53(2) Å, <(N4–H···Cl) 148(2)°.

The crystal structure of salt **8b** (Figure 2) shows a nearly planar core of the molecule (the triazole ring, N4, N5, N6). One face of this core is occupied by the three benzylic substituents, leaving the other face open for the chloride anion which is held in place by two N–H···Cl hydrogen bonds, one of them being very short (*d*(H···Cl) = 2.11(2) Å, *d*(N···Cl) = 3.060(1) Å) and linear and the other one much longer and non-linear. Short N–H···Cl hydrogen bonds have also been found in the crystal structures of related compounds, such as **II**·HCl·CH_3_OH (*d*(H···Cl) = 2.36 Å, *d*(N···Cl) = 3.15 Å [35]) and a 2-phenylamino-1,3,4-thiadiazolium chloride (2.05/3.00 Å [36]). In the packing arrangement of the crystal, the chloride ions are found in anion channels oriented along the 2_1_ screw axis parallel to the crystallographic *a*-axis of the orthorhombic unit cell (space group *P*2_1_2_1_2_1_).

**Figure 3:**
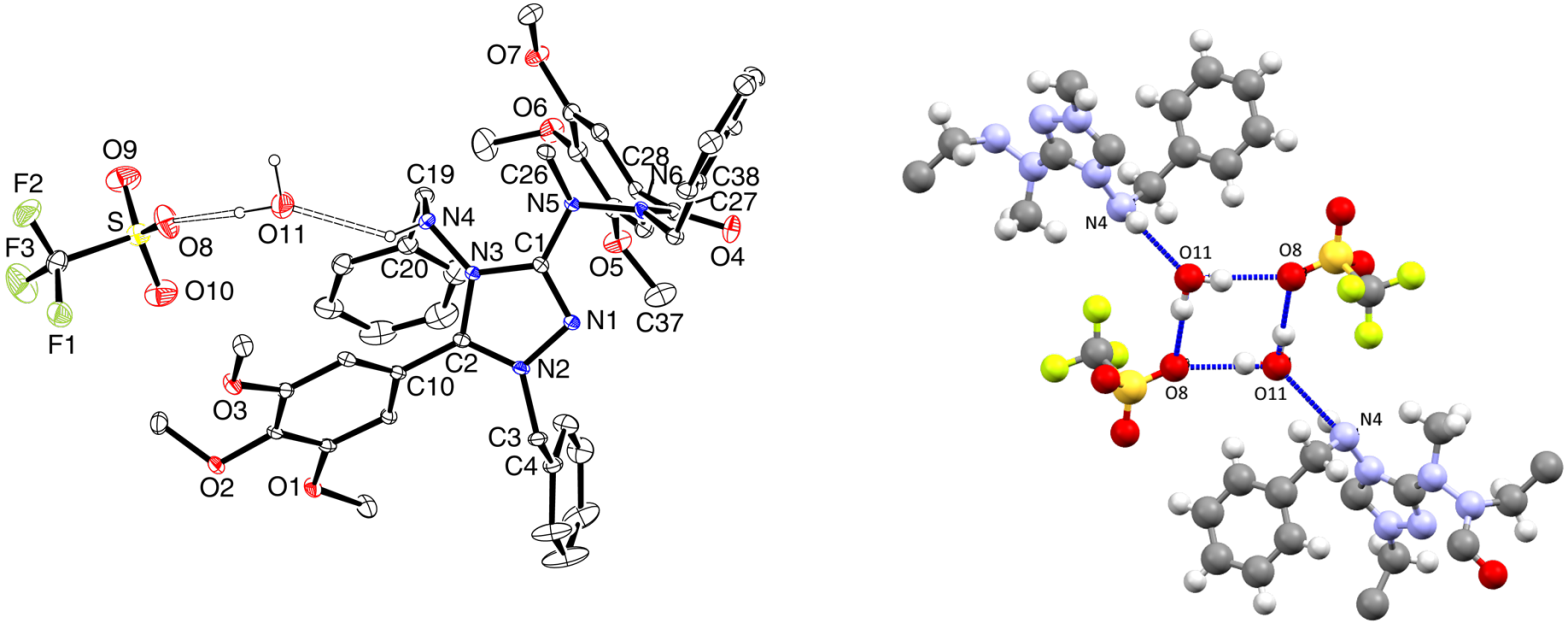
Left: solid-state structure of **9b**·H_2_O (ORTEP plot). Right: centrosymmetric hydrogen-bonded dimer of (CF_3_SO_3_)^−^·H_2_O (Mercury plot); the substituents at the triazole ring are not complete. Selected bond lengths (Å): C1–N1 1.309(3), C1–N3 1.376(3), C1–N5 1.376(3), N1–N2 1.378(3), C2–N2 1.314(3), C2–N3 1.366(3), N3–N4 1.400(3). Hydrogen bonds: N4–H···O11, N4–H 0.89(4) Å, N···O11 2.946(3) Å, H···O11 2.22(4) Å, <(N4–H···O11) 139(3)°; O11–H^b^···O8, O11–H^b^ 1.01 Å, O11···O8 3.023(4) Å, H^b^···O8 2.02 Å, <(O11–H^b^···O8) 170°; O11–H^a^···O8 (1−*x*, 1−*y*, 1−*z*), O11–H^a^ 0.95 Å, O11···O8 2.979(3) Å, H^a^···O8 2.05 Å, <(O11–H^a^···O8) 169°.

The molecular shape of the cation of 1,2,4-triazolium triflate **9b** in the solid state (Figure 3) is similar to that of **8b**. However, the uptake of one water molecule per formula unit has resulted in different hydrogen bond patterns. A centrosymmetric hydrogen-bonded dimer (CF_3_SO_3_·H_2_O)_2_ is present which is connected to two cations through hydrogen bonds with N4–H as the donor and the oxygen atom of water as the acceptor (Figure 3, right).

### Structural and spectroscopic characterization of 1,2,4-triazolium-3-aminides **7**

The bond structure of mesoionic compounds such as **7** can be described as resonance hybrids of quite a number of canonical structures. Depending on the definition of mesoionic compounds, two resonance hybrid formulations are frequently found in the literature: the one displayed in Scheme 1, which assumes the aromatic electron delocalization over the positively charged heterocyclic ring [37], and the one used in the rest of this paper, which is based on a more recent definition [38] and that divides the mesoionic system into two oppositely charged resonance-stabilized moieties (here: an amidinium/amidinate combination). These two moieties are separated by bonds having high single-bond character. The latter concept is supported inter alia by recent theoretical studies using DFT calculations, Natural Bond Orbital analysis and Natural Resonance Theory calculations for mesoionic systems of the 1,3-oxazole, 1,3-diazole and 1,3-thiazole type [39].

**Figure 4:**
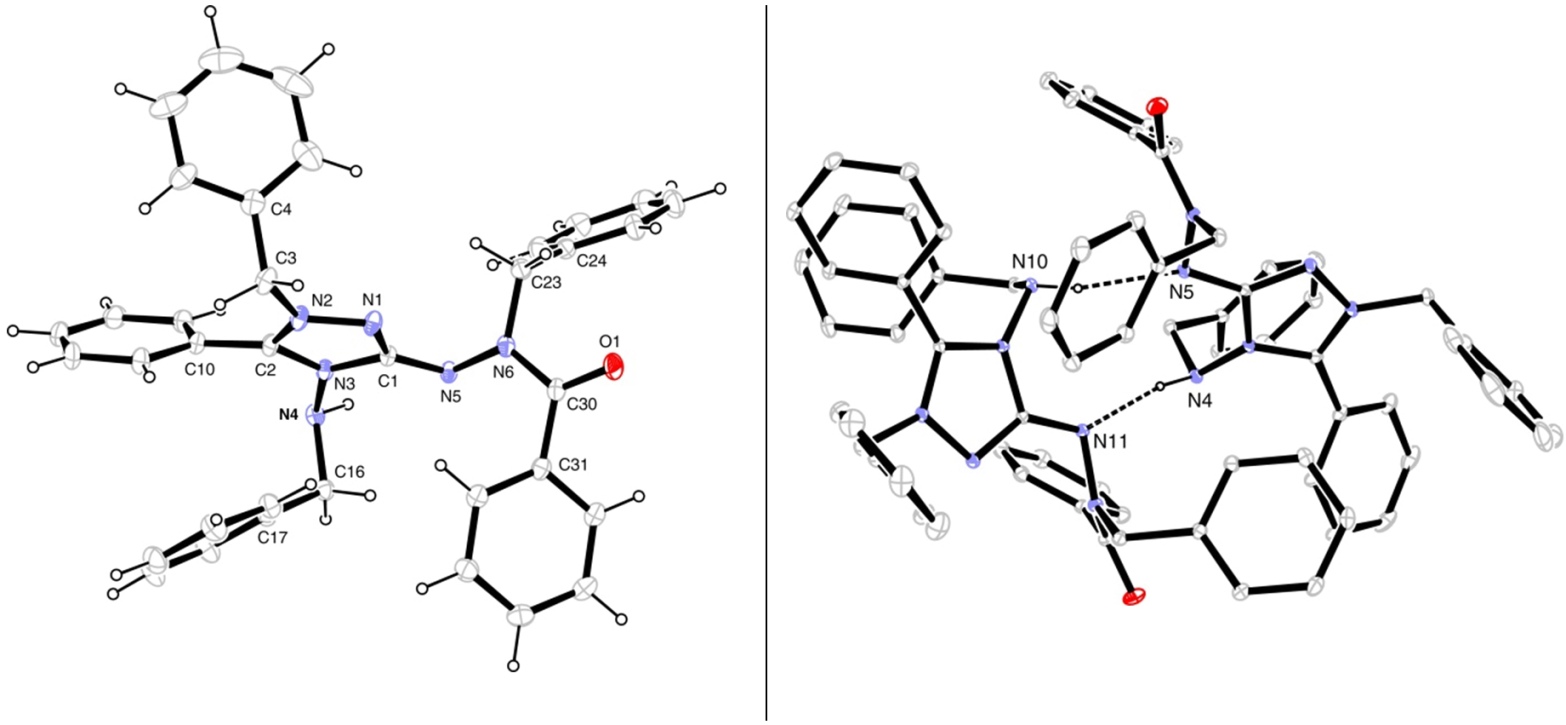
Solid-state structure of mesoionic compound **7a** (ORTEP plot); thermal displacement ellipsoids are drawn at the 30% probability level. Selected bond lengths (Å): C1–N1 1.343(1), C1–N3 1.398(1), C1–N5 1.322(1), N1–N2 1.389(1), C2–N2 1.317(1), C2–N3 1.357(1); selected torsion angles (°): N2–N1–C1–N5 177.7(1), N3–C2–C10–C11 -42.8(2). Right: Hydrogen bonds between two symmetrically not related molecules of **7a** are shown; N10···N5 2.939(2) Å, N10H···N5 2.057(1) Å, <(N10–H···N5) 167.0(2)°; N4···N11 2.969(2) Å, N4H···N11 2.061(1) Å; <(N4–H···N11) 168.7(2)°.

As 1,2,4-triazolium-3-aminides have not yet been studied in depth with respect to their structural and electronic properties, we herein present some relevant data of the 1,2,4-triazolium-hydrazinides **7**. Thus, the molecular structure of **7a** in the solid state was determined by X-ray diffraction analysis and is shown in Figure 4. Two symmetrically independent molecules are present in the triclinic unit cell, being associated by two N–H···N hydrogen bonds in which benzylamino NH bonds act as donors and aminide nitrogen atoms as acceptors.

As expected, the triazolium-aminide moiety in **7a** is planar. The comparison of the bond lengths in this moiety with those in nitron **II** reveals quite similar values for corresponding bonds (Table 2). The data are in agreement with the description of mesoionic compounds **7** as a combination of an amidinium part with delocalized positive charge and an amidinate part incorporating the negative charge [38]. The separation between these two moieties is indicated by the long C–N bond “c” (as compared to the other C–N bonds in the triazole ring) and the endocyclic N–N bond, both of which have a high single-bond character. The influence of the substituents on the triazole ring of **II** and **7a** on the bond lengths is moderate. The bond geometry of the parent 1,2,4-triazolium-3-aminide has been calculated by quantum chemical ab initio methods [40]. The bond length differences are in qualitative agreement with the experimentally determined values for **7a**. On protonation or methylation of the exocyclic aminide nitrogen atom, the mesoionic moiety is converted into a 3-hydrazinyl-substituted 1,2,4-triazolium ion with a delocalized 6π-electron aromatic system. This affects significantly (≥0.22 Å) the three bonds around ring atom C-3, where the two endocyclic bonds are shortened and the exocyclic one is expanded.

**Table 2:** Selected bond lengths (Å) in 1,2,4-triazolium-3-aminides **II** and **7a** and 1,2,4-triazolium salts **8b** and **9b**.

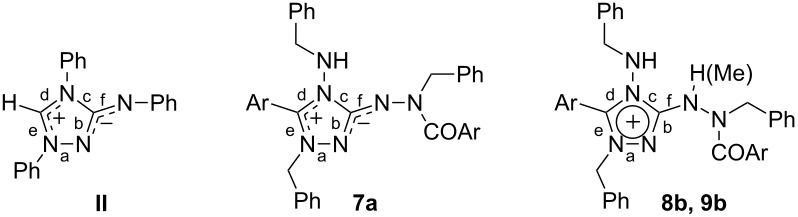				

Bond	**II**^ a^	**7a**	**8b**	**9b**

a	1.40–1.41	1.389(1)	1.388(2)	1.378(2)
b	1.35–1.36	1.343(1)	1.317(2)	1.309(3)
c	1.41–1.43	1.398(1)	1.372(2)	1.376(3)
d	1.32–1.35	1.357(1)	1.352(2)	1.366(3)
e	1.33	1.317(1)	1.319(2)	1.314(3)

f	1.31–1.33	1.322(1)	1.349(2)	1.376(3)

^a^ Lit. [18]; two symmetrically independent molecules.

In the ^13^C NMR spectra of 1,2,4-triazolium-3-aminides **7a–e**, the chemical shifts of ring atoms C-3 and C-5 are of interest (Table 3; for ab initio GIAO-CHF calculations of chemical shifts of the parent 1,2,4-triazolium-3-aminide, see lit. [40]). The C-3 chemical shifts of **7a–e** are equal within 1.14 ppm and are in the typical range for guanidines and guanidinium salts. Notably, the chemical shifts of the amidinate carbon atom C-3 in **7a–d** appear at δ-values that are higher by 2.98**–**7.07 ppm than those of the corresponding neutral guanidine derivatives **6a–d**, in spite of the formal negative charge in the amidinate unit. In contrast, the δ(C-5) values vary strongly with the substituent attached to this carbon atom. Whereas the values for **7a–c** reflect the different interaction of the phenyl, 3,4,5-trimethoxyphenyl and 4-nitrophenyl substituents with the positive charge density at C-5 through mesomeric and inductive effects, the δ(C-5) value in **7d** corresponds to the deshielding α-effect of a methyl group, if one takes the δ-value of the unsubstituted C-5 carbon nucleus in **II** as a reference.

**Table 3:** ^13^C chemical shifts of C-3 and C-5 (triazole ring) of mesoionic compounds **7a–e** and **II**, triazolium salt **8b** and triazole **10b**^a^.

Comp.	R	δ(^13^C) [ppm]	
		C-3 ((CN_3_)^−^)	C-5 ((NCN)^+^)

**7a**	Ph	159.57	120.69
**7b**	3,4,5-(CH_3_O)_3_-C_6_H_2_	159.44	115.62
**7c**	4-NO_2_-C_6_H_4_	159.83	126.39
**7d**	CH_3_	158.69	145.23
**7e**	CH_3_	158.84	144.96
**II**^b^		153.9	133.5
**8b**		152.54	112.92
**10b**		153.60	122.66

^a^Spectra were measured in [*D*_6_]DMSO solution. ^b^Lit. [41].

**Figure 5:**
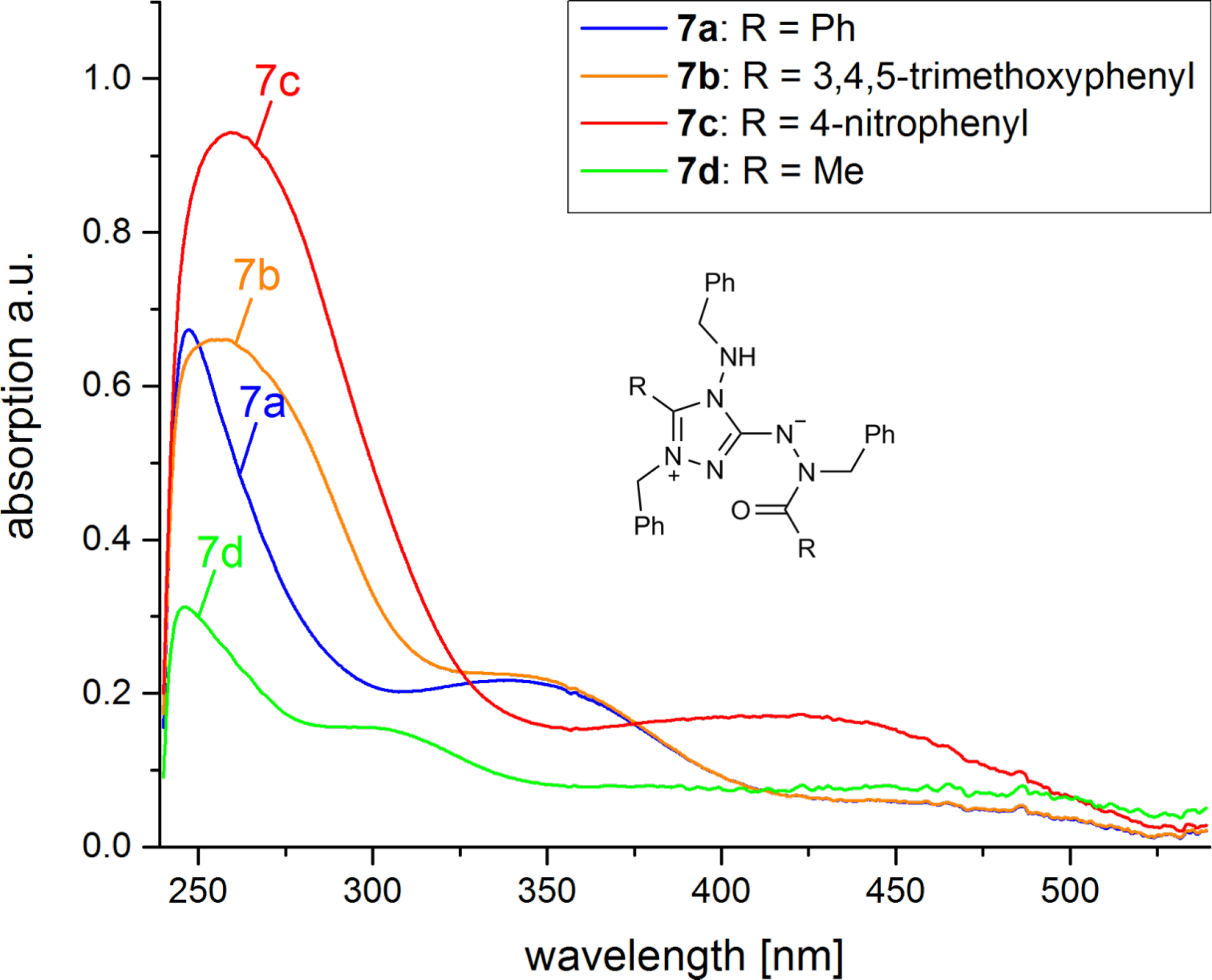
UV–vis spectra of **7a–d** in chloroform (*c* = 0.04 mmol L^−1^); λ_max_ [nm] (ε [L mol^−1^ cm^−1^]): **7a**: 350 (4710); **7b**: 350 (5080); **7c**: 430 (3510); **7d**: 300 (2670).

The UV–vis spectra of **7a–d** are presented in Figure 5. One notes an increasingly bathochromic shift of the long-wavelength absorption maximum in the series R = CH_3_ < phenyl ≈ 3,4,5-trimethoxyphenyl < 4-nitrophenyl. For **7a–c**, this absorption band could result from a charge transfer between the amidinate moiety, representing the HOMO of the mesoionic system, and an unoccupied π-orbital of the C(=O)Ar group.

Upon N-protonation or N-methylation, the mesoionic system loses its betainic character and a 1,2,4-triazolium ion is formed. This is accompanied by the disappearance of the long-wavelength absorption in the electronic spectra, leaving colorless salts **8**.

## Conclusion

We have found that triaminoguanidine, generated from its hydrochloride (TAG-Cl) in aqueous alkaline solution, can be triply acylated to give 1,2,3-tris(acylamino)guanidinium salts only with acyl chlorides that are not easily hydrolyzed. On the other hand, 1,2,3-tris(benzylamino)guanidinium chloride underwent a threefold N-acylation under carefully controlled conditions (typically with a weak base and at room temperature) with aroyl chlorides and with acetyl chloride. Under different conditions (higher temperature, alkaline medium), mesoionic 1,2,4-triazolium-3-hydrazinides were obtained as the major products. The latter compounds were converted into 3-hydrazinyl-1,2,4-triazolium salts by protonation or methylation at the anionic hydrazinide nitrogen atom and into highly substituted and functionalized 1,2,4-triazoles by N-debenzylation through catalytic hydrogenation. Thus, the reaction of triaminoguanidine and its 1,2,3-tribenzyl derivative with acid chlorides gives access to diverse chemical structures which could be considered for further studies, e.g., the biological activity of the triazole-based compounds and the use of threefold symmetrically substituted triaminoguanidines as novel hosts in supramolecular chemistry or as ligands in coordination chemistry.

## Supporting Information

File 1 Experimental procedures, characterization data for synthesized compounds and the data for the X-ray crystal structure determinations.
