# Supplementary material for: Derivatives of the triaminoguanidinium ion, 5. Acylation of triaminoguanidines leading to symmetrical tris(acylamino)guanidines and mesoionic 1,2,4-triazolium-3-aminides
Source: Beilstein J Org Chem. 2017 Mar 22;13:579–88. doi: 10.3762/bjoc.13.57 (PMC5372706; doi:10.3762/bjoc.13.57)
Supplement: File 1 — Experimental procedures, characterization data for synthesized compounds and the data for the X-ray crystal structure determinations. [file Beilstein_J_Org_Chem-13-579-s001.pdf]

Supporting Information

for

Derivatives of the triaminoguanidinium ion, 5. Acylation of triaminoguanidines leading to symmetrical tris(acylamino)guanidines and mesoionic 1,2,4-triazolium-3-aminides

Jan Szabo, Julian Greiner and Gerhard Maas*

Address: Institute of Organic Chemistry I, Ulm University, Albert-Einstein-Allee 11, D-89081 Ulm, Germany

Email: Gerhard Maas* - gerhard.maas@uni-ulm.de

*Corresponding author

For part 4 in this series, see [1].

Experimental procedures, characterization details for synthesized compounds and data of the X-ray crystal structure determinations

Table of contents

Experimental procedures and characterization of compounds S2

X-ray crystal structure determinations S21

References S22

1. Experimental details and characterization of compounds

1.1. General information

^{1}H and ^{13}C NMR spectra were recorded on Bruker Avance 400 (^{1}H: 400.13 MHz; ^{13}C: 100.62 MHz, ^{19}F: 376.47 MHz) and Bruker Avance 500 spectrometers (^{1}H: 500.13 MHz, ^{13}C: 125.76 MHz); δ values are reported in ppm and coupling constants are given in Hertz (Hz). The signal of the solvent was used as an internal standard: ^{1}H spectra: $\delta((CH_3)_2SO)$ = 2.50 ppm; ^{13}C spectra: $\delta((CD_3)_2SO)$ = 39.43 ppm. ^{19}F spectra were referenced to external C_6F_6 (δ = −164.90 ppm). The NMR spectra were measured at 298 ± 2 K, if not stated otherwise. When necessary, ^{13}C signal assignments were derived from C,H COSY, HSQC and HMBC spectra. IR spectra: Bruker Vector 22; wavenumbers [cm^{-1}] and intensities (vs = very strong, s = strong, m = medium, w = weak, br = broad) are given. Elemental analyses: elementar vario MICRO cube. Mass spectra: Bruker Daltonics REFLEX III (MALDI-TOF spectra; matrix: *trans*-2-(3-(4-*tert*-butylphenyl)-2-methyl-2-propenylidene)malononitrile); Bruker solariX (MALDI-TOF and ESI HRMS spectra). Melting points were determined with a Büchi Melting Point B-540 apparatus.

1.2. Materials

1,2,3- (or *N*,*N*',*N*'')-triaminoguanidinium chloride (**1**) [1] and 1,2,3-(or *N*,*N*',*N*'')-tris(benzylamino)guanidinium chloride (**4**) [2] were prepared by published procedures. All other chemicals were purchased commercially or were available in the laboratory. Acetyl chloride, benzoyl chloride and methyl trifluoromethanesulfonate were freshly distilled before use.

1.3. Synthetic procedures

1.3.1. 1,2,3-Tris(3,4,5-trimethoxybenzamido)guanidinium chloride (3)

A solution of 1,2,3-triaminoguanidinium chloride (**1**, 680 mg, 4.84 mmol) in aqueous sodium hydroxide (5 M, 3 mL) was cooled to 10 °C and a solution of 3,4,5-trimethoxybenzoyl chloride (**2b**, 3.47 g, 15.06 mmol) in 1,4-dioxane (20 mL) was gradually added during 2 h. The mixture was stirred for additional 30 min at room temperature, then brought to pH 4 with concentrated hydrochloric acid, whereupon a colorless solid separated. After evaporation of the volatiles, the solid residue was dispersed in water (5 mL) and kept in an ultrasonic bath for 2 h. The powdery solid was isolated by filtration, washed with water followed by diethyl ether and freeze-dried to furnish 2.98 g (4.12 mmol, 85%) of the colorless product, m. p. 198.6–199.6 °C. – ^{1}H NMR (500.16 MHz, $(CD_3)_2SO$): δ = 3.72 (s, 3 H, *p*-OCH_3), 3.86 (s, 6 H, *m*-OCH_3), 7.38 (s, 2 H, CH_{Ar}), 10.70 (s, 1 H, C^+–NH), 11.05 (s, 1 H, NHCO) ppm. – ^{13}C NMR (125.76 MHz, $(CD_3)_2SO$): δ = 56.26 (3-OCH_3), 60.26 (4-OCH_3), 105.85 (CH_{Ar}), 127.04 (C-1_{Ar}), 140.78 (C-4_{Ar}), 152.66 (C-3_{Ar}), 158.48 (C^+N_3), 166.11 (C=O) ppm. – IR (KBr): ν = 3420 (very broad, w), 3238 (br, m), 2944 (m), 2839 (w), 1664 (m), 1586 (s), 1500 (m), 1446 (m), 1415 (m), 1337 (s), 1237 (m), 1128 (vs), 999 (m) cm^{-1}. – Anal. calcd. for $C_{31}H_{39}N_6O_{12}$ (723.13): C 51.49, H 5.44, N 11.62; found: C 51.45, H 5.56, N 11.67.

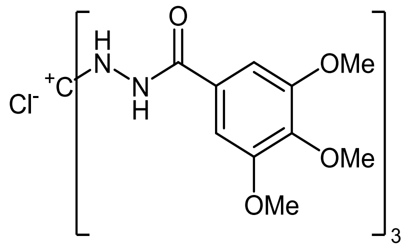

1.3.2. 1,2,3-Tris(4-fluorobenzylidenamino)guanidinium chloride (no formula number)

By analogy to a published procedure [2], 1,2,3-triaminoguanidinium chloride (**1**, 9.51 g, 67.65 mmol) was dissolved in ethanol-water (200 mL, 1:1 v/v) at 85 ^{o}C. A solution of 4-fluorobenzaldehyde (25.30 g, 203.85 mmol) in ethanol (50 mL) was added dropwise. The mixture was kept with stirring at reflux temperature for 1 h, during which time an off-white precipitate appeared. After cooling, the solid was isolated by filtration, washed with ethyl acetate and dried at 50 ^{o}C/0.05 mbar. Yield: 30.72 g (66.95 mmol, 99%); m. p. 244.7–245.0 ^{o}C. – ^{1}H NMR (400.13 MHz, $(CD_3)_2SO$): δ = 7.38 (t, $J_{H,H}$ = 8.8 Hz, 2 H, H_{Ar}), 8.09 (dd, $J_{H,H}$ = 8.8 Hz, $J_{H,F}$ = 3.2 Hz, 2 H, H_{Ar}), 8.80 (s, 1 H, N=CH), 12.29 (s, 1 H, C^+N_3) ppm. – ^{13}C NMR (100.61 MHz, $(CD_3)_2SO$): δ = 116.02 (d, $^{2}J_{C,F}$ = 21.9 Hz, *m*-CH_{Ar}), 129.78 (d, $^{4}J_{C,F}$ = 2.8 Hz, *i*-C_{Ar}), 130.65 (d, $^{3}J_{C,F}$ = 8.6 Hz, *o*-CH_{Ar}), 149.19 (C^+N_3), 150.07 (N=CH),

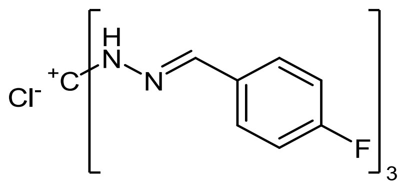

163.81 (d, $^1J_{C,F}$ = 249.3 Hz, C_{Ar}-F) ppm. –^{19}F NMR (376.47 MHz, $(CD_3)_2SO$): δ = -108.80 ppm. – IR (KBr): ν = 3360 (w), 1647 (s), 1507 (s), 1427 (w), 1322 (m), 1231 (s), 1155 (m), 1089 (m), 972 (w), 840 (m), 800 (w) cm^{-1}. – Anal. calcd. for $C_{22}H_{28}ClF_3N_6$ (458.87): C 57.58, H 3.95, N 18.31; found C 57.27, H 3.97, N 18.34.

1.3.3. 1,2,3'-Tris(4-fluorobenzylamino)guanidinium chloride (5)

Synthesis by catalytic hydrogenation of 1,2,3-tris(4-fluorobenzylidenamino)-guanidinium chloride (section 1.3.2; 10.00 g, 21.79 mmol) with Pd/C (10%, 160 mg) in methanol (250 mL) according to lit. [2]. – Yield: 3.20 g (17.64 mmol, 81%); m. p. 148.5–155.8 °C (heating rate 2 °C/min). – ^{1}H NMR (400.13 MHz, $(CD_3)_2SO$): δ = 3.72 (d, $^3J_{H,H}$ = 4.7 Hz, 6 H, CH_2), 5.43 (t, $^3J_{H,H}$ = 4.9 Hz, 3 H, N*H*CH_2), 7.05–7.15 (m, 6 H, H_{Ar}), 7.31–7.37 (m, 6 H, H_{Ar}), 8.76 (s, 3 H, C^+NH) ppm. – ^{13}C NMR (100.61 MHz, $(CD_3)_2SO$): δ = 53.47 (CH_2), 114.82 (d, $^2J_{C,F}$ = 21.2 Hz, *m*-CHAr), 131.00 (d, $^3J_{C,F}$ = 8.1 Hz, *o*-CH_{Ar}), 133.28 (d, $^4J_{C,F}$ = 2.9 Hz, *i*-C_{Ar}), 157.03 (C^+N_3) ppm, 161.53 (d, $^1J_{C,F}$ = 243.1 Hz). – ^{19}F ($CDCl_3$): δ = -116.60 ppm. – IR (KBr): ν = 3420 (br, m), 3339 (m), 3204 (s), 1653 (s), 1607 (m), 1512 (s), 1230 (s), 1159 (w), 830 (s) cm^{-1}. – Anal. calcd. for $C_{22}H_{24}ClF_3N_6$ (464.92): C 56.84, H 5.20, N 18.08; calcd. for M × 0.64 H_2O: C 55.46, H 5.35, N 17.64; found: C 55.46; H 5.05, N 17.51.

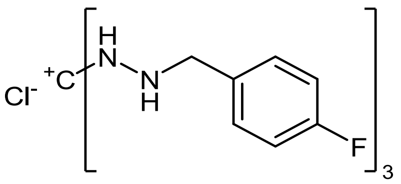

*1.3.4. Acylation of triaminoguanidinium salt **4** to give guanidines **6** selectively*

1.3.4.1. 1,2,3-Tris(*N*-benzyl-2-benzamido)guanidine (6a) and betaine 7a

1,2,3-Tris(benzylamino)guanidinium chloride (**4**, 2.00 g, 4.87 mmol) was dissolved in chloroform (50 mL), sodium carbonate (3.50 g, 33.02 mmol) was added, and the suspension was heated at reflux while stirring vigorously. A solution of benzoyl chloride (**2a**, 2.3 mL, 19.96 mmol) in chloroform (50 mL) was gradually added during one hour. After the addition the suspension was stirred for additional three hours. After cooling to room temperature, the solid component was filtered off, the mother liquor was evaporated, and the yellow greasy residue was triturated with diethyl ether. The

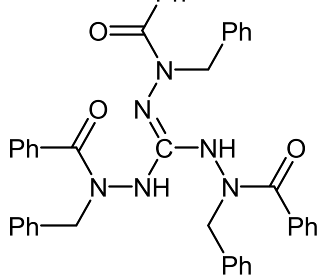

resulting colorless solid was filtered off and briefly treated with boiling acetone (20 mL). The suspension was filtered hot and the colorless solid so obtained was dried (50 °C/0.02 mbar); yield: 2.93 g (4.27 mmol, 88%), m. p. 233.8–234.1 °C. – IR (KBr): ν = 3272 (m), 3059 (w), 3030 (w), 1649 (s), 1621 (s), 1521 (m), 1496 (m), 1440 (s), 1407 (m), 1356 (m), 1286 (m), 1155 (w), 1076 (w), 1028 (w), 986 (m), 915 (w), 777 (w), 730 (m), 698 (s), 633 (w) cm^{-1}. – ^1H NMR (500.16 MHz, $(CD_3)_2SO$): δ = ~2.7–3.4 and 4.8–5.3 (signals in coalescence, 6 H, CH_2), 6.9–7.5 (m, 30 H, CH_{Ph}), ~9.1–9.5 (signals in coalescence, 2 H, NH) ppm. – ^{13}C NMR (125.76 MHz, $(CD_3)_2SO$; many signals more or less broadened at *T* = 298 K): δ = 49.70, 49.90, 50.47 (all CH_2); 126.45, 126.92, 127.01, 127.16, 127.28, 127.53, 127.68, 128.05, 128.09, 128.20, 128.55, 129.26, 129.43 (all CH_{Ph}); 135.00, 135.45, 135.69, 135.98, 136.49, 136.91, 137.38, 137.58 (all C_{Ph}); 153.11 (C=N); 170.34, 171.91, 172.20, 173.09 (all C=O) ppm. – MS (ESI): *m/z* = 687.31 $[M + H]^+$. – Anal. calcd. for $C_{43}H_{38}N_6O_3$ (686.82): C, 75.20; H, 5.58; N, 12.24; found: C, 75.33; H, 5.48; N, 12.22.

Under identical conditions for the synthesis, but with a different workup, betaine **7a** was isolated in 7% yield (see section 1.3.5.1).

1.3.4.2. 1,2,3-Tris(*N*-benzyl-3,4,5-trimethoxybenzamido)guanidine (6b)

1,2,3-Tris(benzylamino)guanidinium chloride (**4**, 410 mg, 1.00 mmol) was dissolved in chloroform (20 mL) and sodium carbonate (760 mg, 7.17 mmol) was added.

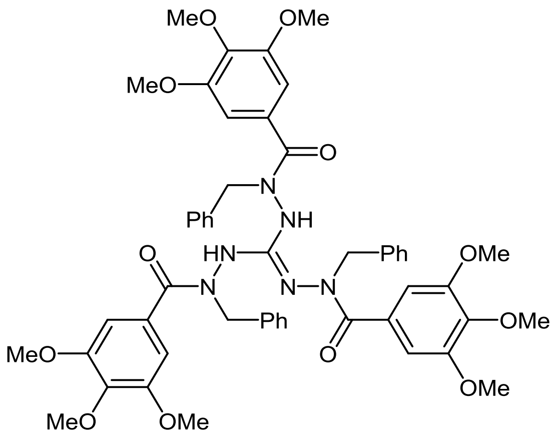

During 30 min a solution of 3,4,5-trimethoxybenzoyl chloride (**2b**, 695 mg, 3.01 mmol) in chloroform (10 mL) was slowly added, then the reaction mixture was stirred vigorously for additional 18 h and the solid component was filtered off. The mother liquor was evaporated to dryness, and the solid residue was recrystallized from isopropyl alcohol and dried (50 °C/0.02 mbar) to furnish a colorless solid (450 mg, 0.47 mmol, 47%), m. p. 115.1–115.6 °C. – IR (KBr): ν = 3262 (br, m), 2939 (m), 2835 (w), 1655 (s), 1620 (s), 1584 (s), 1502 (s), 1456 (s), 1413 (s), 1329 (s), 1236 (s), 1127 (s), 1003 (m), 925 (w), 884 (w), 847 (w), 702 (m), 630 (m) cm^{-1}. – ^1H NMR (400.13 MHz, $(CD_3)_2SO$, 298 K): see Fig. S1. – ^{13}C

NMR (100.61 MHz, $(CD_3)_2SO$, 298 K): broadened signals or groups of broadened signals at δ = 55.8, 59.9, 128.2, 138.7, 152.4 ppm; C=O signal not visible. – MS (ESI): *m*/*z* = 957.40 $[M + H]^+$. – Anal. calcd. for $C_{52}H_{56}N_6O_{12}$ (957.05): C, 65.26; H, 5.90; N, 8.78; found: C, 65.37; H, 5.98; N, 9.05.

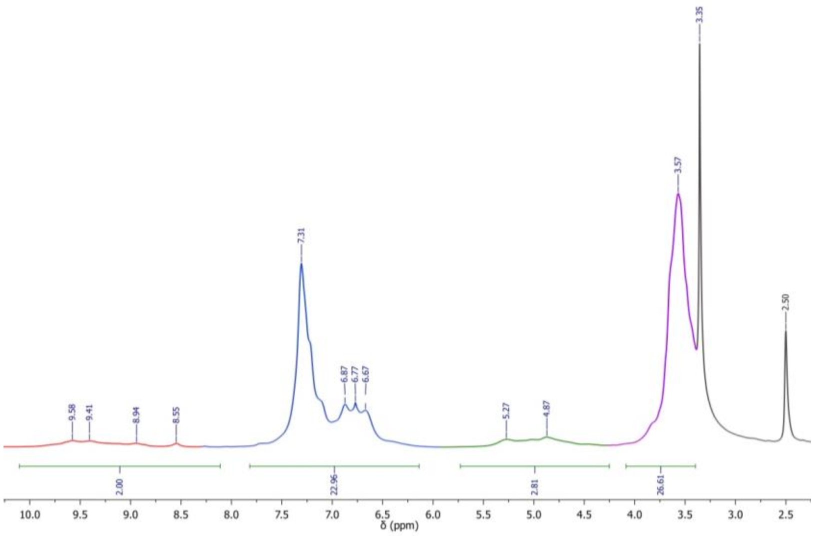

Figure S1: ^{1}H NMR spectrum of **6b** ($(CD_3)_2SO$, 400.13 MHz, 298 K) with signals for $(CH_3)_2SO$ (δ = 2.50 ppm) and water (3.35 ppm). Four signal groups are in coalescence: the amino protons (red), the aromatic protons (blue), the methylene protons (one half in the region marked in green) and the methyl protons plus the other half of the methylene protons (purple) (from left to right).

1.3.4.3. 1,2,3-Tris(*N*-benzyl-4-nitrobenzamido)guanidine (6c)

Variation 1 (at room temperature). 1,2,3-Tris(benzylamino)guanidinium chloride (**4**, 0.20 g, 0.49 mmol) and Na_2CO_3 (0.34 g, 3.21 mmol) were suspended in chloroform

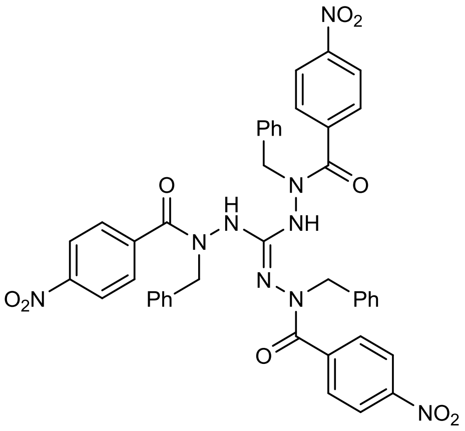

(20 mL) under an argon atmosphere. A solution of 4-nitrobenzoyl chloride (**2c**, 0.26 g, 1.40 mmol) in chloroform (10 mL) was gradually added during 30 min and the reaction mixture was stirred for additional 30 min. The solid components of the mixture were filtered off with suction and the mother liquor was treated with aqueous NaOH (1 M, 10 mL). After phase separation, the organic layer was washed with water and dried ($MgSO_4$). The solvent was evaporated and the solid residue was dissolved in as little chloroform as possible. This solution was added drop by drop to diethyl ether, the obtained precipitate was isolated by suction

filtration, washed with diethyl ether and dried at 50 °C/0.05 mbar. Yield of **6c**: 0.15 g (0.18 mmol, 38%).

Variation 2 (at 70 °C). 1,2,3-Tris(benzylamino)guanidinium chloride (**4**, 2.00 g, 4.87 mmol) and K_2CO_3 (3.00 g, 21.71 mmol) were added to acetonitrile (50 mL) under an argon atmosphere. The mixture was heated at reflux temperature, 4-nitrobenzoyl chloride (**2c**, 3.60 g, 19.40 mmol) was added and the mixture was kept with stirring at 70 °C for additional 3 hours. The solid components of the mixture were removed by suction filtration (the mother liquor contains betaine **7c**), and suspended in water (30 mL). After extraction with chloroform (2 × 20 mL), the combined organic phases were dried ($MgSO_4$) and the volatiles were evaporated. Product isolation as described for variation 1 furnished 0.85 g (1.03 mmol, 21%) of **6c**. With a different workup, betaine **7c** (41%) was isolated (see section 1.3.5.3).

Data for **6c**: m. p. 197.0–197.6 °C. – ^{1}H NMR (500.16 MHz, $(CD_3)_2SO$, 359 K): δ = 3.40–5.30 (coalescing signals, 6 H, CH_2), 7.00–7.60 (several broadened signals, 21 H, H_{Ar}), 7.80–8.10 (coalescing signals, 6 H, CH_{Ar}), 9.10–9.25 (coalescing signals, 2 H, NH) ppm. – ^{13}C NMR (125.76 MHz, $(CD_3)_2SO$, 298 K): δ = 50.06, 50.27, 54.80 (all CH_2); 122.20, 122.27, 122.66, 122.94, 126.91, 127.05, 127.39, 127.54, 127.92, 128.15, 128.20, 128.25, 128.40, 128.46, 128.76 (all CH_{Ar}); 134.75, 135.10, 135.85, 135.92, 136.09, 137.05, 141.66, 143.42, 147.04, 147.44, 147.89 (all C_{Ar}); 156.85 (CN_3), 167.57, 168.03, 170.07 (all C=O) ppm. The majority of signals is broadened due to dynamic processes within the molecules. – HRMS (ESI): *m/z* = 822.2625 (calcd. 822.2631 for $C_{43}H_{36}N_9O_9$, $[M + H]^+$). – IR (KBr): ν = 3288 (br, m), 3109 (w), 3066 (w), 3032 (w), 2937 (w), 2862 (w), 1661 (s), 1602 (s), 1524 (vs), 1437 (m), 1411 (m), 1349 (vs), 1315 (m), 1290 (m), 1108 (w), 984 (w), 857 (s), 732 (m), 704 (s) cm^{-1}. – Anal. calcd. for $C_{43}H_{35}N_9O_9$ (821.81): C 62.85, H 4.29, N 15.34; calcd. for M × 1 H_2O: C 61.50, H 4.44, N 15.01; found: C 61.44, H 4.37, N 15.12.

1.3.4.4. 1,2,3-Tris(*N*-benzylacetamido)guanidine (6d)

This compound was prepared as described for **6b** (section 1.3.4.2) from salt **4** (543 mg, 1.32 mmol) and acetyl chloride (0.48 mL, 6.7 mmol) in chloroform (20 mL) in the presence of Na_2CO_3 (1.00 g, 9.44 mmol). Crystallization from acetonitrile yielded **6d**

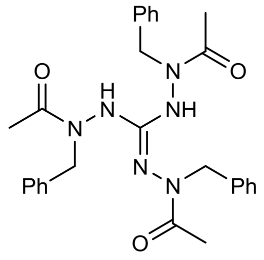

as a colorless solid (319 mg, 0.64 mmol, 49%); m. p. 195.6–197.2 °C (dec.). – ^{1}H NMR (500.16 MHz, $(CD_3)_2SO$, 351 K): δ = 1.85 (s, 3 H, CH_3), 1.90–2.10 (coalescing signals, 6 H, CH_3); ~3.7, 4.4 and 5.0 (3 very broad coalescing signals, 6 H, CH_2); 7.20–7.40 (m, 15 H, H_{Ph}), 8.57 (broadened signal, 2 H, NH) ppm. – ^{13}C NMR (125.76 MHz, $(CD_3)_2SO$, 298 K): δ = 20.39, 20.56, 21.03 (all CH_3); 50.15 (CH_2), 51.60 (CH_2); 126.82, 127.17, 127.26, 127.53, 127.61, 128.03, 128.28, 128.71, 128.93, 129.24 (all CH_{Ar}); 135.88, 136.99, 137.91 (all C_{Ar}); 153.96/154.14 (CN_3); 170.11, 172.94, 173.11 (all C=O) ppm. Several ^{13}C signals are broadened. – HRMS (ESI): *m/z* = 501.2606 (calcd. 501.2609 for $C_{28}H_{33}N_6O_3$, $[M + H]^+$), 1001.5167 (calcd. 1001.5145 for $C_{56}H_{65}N_{12}O_6$, $[2M + H]^+$). – IR (KBr): ν = 3305 (m), 3167 (m), 3032 (m), 2926 (w), 2886 (w), 1685 (s), 1661 (s), 1616 (s), 1527 (m), 1450 (m), 1415 (s), 1361 (m), 1266 (m), 985 (m), 741 (m), 700 (m) cm^{-1}. – Anal. calcd. for $C_{28}H_{32}N_6O_3$ (500.60): C 67.18, H 6.44, N 16.79; found: 67.14, H 6.53, N 16.83.

*1.3.5. Acylation of triaminoguanidinium salts **4** and **5** leading to betaines **7a–e** selectively*

1.3.5.1. 2-Benzoyl-2-benzyl-1-(1-benzyl-4-(benzylamino)-5-phenyl-1*H*-1,2,4-triazol-4-ium-3-yl)hydrazin-1-ide (7a)

Variation 1. Under an argon atmosphere, 1,2,3-tris(benzylamino)guanidinium chloride (**4**, 2.00 g, 4.87 mmol) was suspended in hot acetonitrile (50 mL). Aqueous NaOH (5

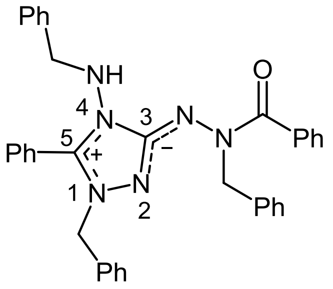

M, 15 mL) and benzoyl chloride (**2a**, 3.5 mL, 30.1 mmol) were added and the solution was kept with stirring at reflux for 3 h. After cooling to room temperature, water (30 mL) was added, and the solution was extracted with ethyl acetate (30 mL). The organic layer was separated and washed with aqueous NaOH (1 M, 20 mL) followed by water. After drying ($MgSO_4$), the solvent was evaporated and the solid residue was

taken up in hot ethyl acetate (25 mL). The insoluble residue was separated by suction filtration. From the filtrate, **7a** crystallized on cooling and was isolated. After drying at 40 °C/0.05 mbar, the product was obtained as a yellowish powdery solid (1.30 g, 2.30 mmol, 47%), m. p. 138.5–139.8 °C. – Anal. calcd. for $C_{36}H_{32}N_6O$ (564.69): C, 76.57, H, 5.71, N, 14.88; found: C, 76.57, H, 5.78, N, 14.88. – IR (KBr): ν = 3061 (w), 3029 (w), 2931 (w), 1594 (s), 1573 (s), 1525 (m), 1494 (m), 1451 (m), 1356 (w), 1266 (m), 1148 (w), 1078 (w), 1029 (w), 699 (s) cm^{-1}. – ^1H NMR (500.16 MHz, $(CD_3)_2SO$): δ = 3.60 (s, 2 H, NHCH_2), 4.92 (s, 2 H, CO-NCH_2), 5.03 (s, 2 H, N^+CH_2), 6.32 (d, *J* = 5.6 Hz, 2 H, H_{Ph}), 6.70–6.78 (m, 2 H_{Ph} and NH), 6.87–6.92 (m, 2 H, H_{Ph}), 7.00–7.07 (m, 3 H, H_{Ph}), 7.15–7.20 (m, 1H, H_{Ph}), 7.23–7.40 (m, 8 H, H_{Ph}), 7.41–7.50 (m, 5 H, H_{Ph}), 7.53–7.62 (m, 2 H, H_{Ph}) ppm. – ^{13}C NMR (100.61 MHz, $(CD_3)_2SO$): δ = 49.16 (CO-N-CH_2), 50.93 ($NHCH_2$), 52.47 (N^+CH_2), 120.69 (C_{Ph}); 126.32, 126.50, 127.01, 127.05, 127.20, 127.76, 127.84, 127.88, 128.42, 128.60, 129.18, 129.29, 131.07 (all CH_{Ph}); 135.21, 135.92, 138.51, 139.12 (all C_{Ph}); 146.01 (N^+=C_{Ph}), 159.57 (CN_3), 169.13 (C=O) ppm. – HRMS (ESI): *m/z* = 565.2707 (calcd. 565.2710 for $C_{36}H_{33}N_6O$ $[M + H]^+$). – Anal. calcd. for $C_{36}H_{32}N_6O$ (564.69): C, 76.57; H, 5.71; N, 14.88; found: C, 76.60; H, 5.81; N, 14.88.

1.3.5.2. 2-Benzyl-1-(1-benzyl-4-(benzylamino)-5-(3,4,5-trimethoxyphenyl)-1*H*-1,2,4-triazol-4-ium-3-yl)-2-(3,4,5-trimethoxybenzoyl)hydrazin-1-ide (7b)

Method A. Under an argon atmosphere, 1,2,3-tris(benzylamino)guanidinium chloride (**4**, 1.56 g, 3.80 mmol) was suspended in acetonitrile (50 mL) at 70 °C. After addition of aqueous NaOH (5 M, 15 mL) and 3,4,5-trimethoxybenzoyl chloride (3.30 g, 14.31 mmol), the solution was kept with stirring at 70 °C for 3 hours.

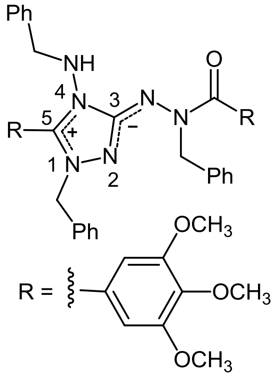

Method B. Under an argon atmosphere, 1,2,3-tris(*N*-benzyl-3,4,5-trimethoxybenzamido)guanidine (**6b**, 110 mg, 0.115 mmol) was dissolved in acetonitrile (10 mL). After addition of aqueous NaOH (5 M, 0.8 mL), the solution was kept with stirring at 70 °C for 3 h.

Workup for both methods: Water was added to the clear yellow solution and the mixture was extracted with two portions of ethyl acetate. The combined extracts were dried ($MgSO_4$), the volatiles were evaporated and the solid residue was recrystallized from

ethanol. After drying at 40 °C/0.05 mbar, the product was obtained as a yellow powdery solid (from method A: 2.37 g, 3.18 mmol, 84%; from method B: 50 mg (67 μmol, 58%); m. p. 185.2–186.0 °C. – IR (KBr): ν = 3446 (br, w), 2939 (m), 1576 (s), 1501 (s), 1456 (m), 1412 (m), 1367 (w), 1328 (w), 1299 (w), 1239 (m), 1125 (s), 1003 (m), 837 (w), 751 (w), 700 (m) cm^{-1}. – ^1H NMR (400.13 MHz, $(CD_3)_2SO$): δ = 3.47 (s, 6 H, OCH_3), 3.51 (s, 3 H, OCH_3), 3.68 (s, 9 H, OCH_3), 3.74 (s, 2 H, NHC*H*$_2$), 4.91 (s, 2 H, CO-NCH_2), 5.06 (s, 2 H, N^+C*H*$_2$), 6.02 (s, 2 H, N^+=C-CH_{Ar}), 6.40 (d, *J* = 7.3 Hz, 2 H, H_{Ph}), 6.65 (broadened s, 1 H, NH), 6.87 (t, *J* = 7.4 Hz, 2 H, H_{Ph}), 6.94 (s, 2 H, CO-CH_{Ar}), 7.01 (t, *J* = 7.4 Hz, 1 H, H_{Ph}), 7.12 (d, *J* = 6.8 Hz, 2 H, H_{Ph}), 7.20 (t, *J* = 7.3 Hz, 1 H, H_{Ph}), 7.30 (t, *J* = 7.4 Hz, 2 H, H_{Ph}), 7.35–7.39 (m, 3 H, H_{Ph}), 7.50 (d, *J* = 7.4 Hz, 2 H, H_{Ph}) ppm. – ^{13}C NMR (125.76 MHz, $(CD_3)_2SO$): δ = 49.66 (CO-N*C*H_2), 51.13 ($NHCH_2$), 52.85 (N^+CH_2), 55.71 (2 OCH_3), 55.86 (2 OCH_3), 59.89 (1 OCH_3), 60.16 (1 OCH_3), 105.13 (CO-*C*H_{Ar}), 107.10 (CH_{Ar}), 115.62 (C_{Ph}); 126.49, 126.57, 126.66, 127.07, 127.68, 127.97, 128.14, 128.71, 129.22 (all CH_{Ph}); 133.69 (CO-*C*$_{Ar}$), 135.69 (C_{Ph}), 136.12 (C_{Ph}), 138.20 (*C*-OMe), 139.18 (C_{Ph}), 139.68 (1 *C*-OMe), 145.82 (N^+=C), 152.05 (2 *C*-OMe), 152.38 (2 *C*-OMe), 159.44 (CN_3), 168.72 (C=O) ppm. – MS (MALDI-TOF): *m*/*z* = 549.26 $[M + H – ArCO]^+$, 639.26 $[M + H – PhN]^+$, 745.33 $[M + H]^+$, 1490.66 $[2M + H]^+$. – Anal. calcd. for $C_{42}H_{44}N_6O_7$ (744.85): C, 67.73; H, 5.95; N, 11.28; found: C, 67.44; H, 5.93; N, 11.22.

1.3.5.3. 2-Benzyl-1-(1-benzyl-4-(benzylamino)-5-(4-nitrophenyl)-1*H*-1,2,4-triazol-4-ium-3-yl)-2-(4-nitrobenzoyl)hydrazin-1-ide (7c)

Method A. Under an argon atmosphere, 1,2,3-tris(benzylamino)guanidinium chloride (**4**, 2.00 g, 4.87 mmol) and 4-nitrobenzoyl chloride (**2c**, 3.60 g, 19.40 mmol) were dissolved in acetonitrile (50 mL) and heated at reflux. Potassium carbonate (3.0 g, 21.7 mmol) was quickly added and the reaction mixture was kept with stirring at 70 °C for additional 3 h, whereby the liquid phase gradually turned orange. The solid components were filtered off and water was added to the clear orange solution, which was then extracted with several portions of ethyl acetate. The combined organic extracts were dried ($MgSO_4$) and the solvent was removed. The orange residue was briefly extracted with hot methanol (20 mL), the undissolved solid was separated by suction filtration and dried (50

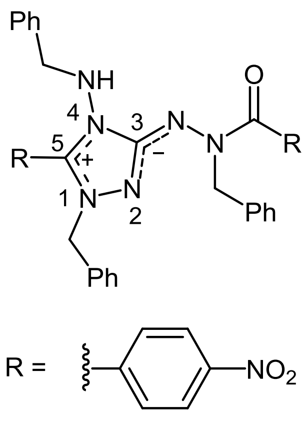

°C/0.05 mbar). An orange solid was obtained (1.31 g, 1.99 mmol, 41%), m. p. 156.2–157.2 °C.

Method B. Under an argon atmosphere, 1,2,3-tris(*N*-benzyl-4-nitrobenzamido)guanidine (**6c**, 100 mg, 0.12 mmol) was dissolved in acetonitrile (10 mL), aqueous NaOH (5 M, 1.2 mL) was added, and the stirred solution was heated at 70 °C for 3 h. After cooling to room temperature, water was added and the clear orange solution was extracted twice with ethyl acetate (2 × 5 mL). The combined extracts were dried ($MgSO_4$) and evaporated to dryness. The residue was treated with hot methanol (5 mL) and the undissolved solid was isolated by suction filtration and dried at 50 °C/0.05 mbar; yield: 40 mg (0.06 mmol, 50%). This product could not be obtained analytically pure, however.

IR (KBr): ν = 3420 (br, w), 3107 (w) 1634 (s), 1589 (s), 1520 (s), 1494 (w), 1452 (w), 1421 (w), 1348 (s), 1288 (w), 1236 (w), 1106 (w), 974 (w), 855 (m), 750 (w), 701 (m) cm^{-1}. – ^1H NMR (500.16 MHz, $(CD_3)_2SO$): δ = 3.67 (s, 2 H, $NHCH_2$), 4.92 (s, 2 H, CO-NCH_2), 5.04 (s, 2 H, N^+CH_2), 6.02 (d, *J* = 7.4 Hz, 2 H, CH_{Ph}), 6.45 (s, 1 H, NH), 6.85 (t, *J* = 7.6 Hz, 2 H, H_{Ph}), 7.06/8.12 (AA'BB' system, ^3J = 8.8 Hz, 4 H, N^+=C-C_6H_4), 7.06 (s, 3 H, H_{Ph}), 7.25 (t, *J* = 7.3 Hz, 1 H, H_{Ph}), 7.35 (t, *J* = 7.5 Hz, 2 H, H_{Ph}), 7.38–7.45 (m, 3 H, H_{Ph}), 7.48 (d, *J* = 7.4 Hz, 2 H, H_{Ph}), 7.77/8.18 (AA'BB' system, ^3J = 8.6 Hz, 4 H, CO-C_6H_4) ppm. – ^{13}C NMR (125.76 MHz, $(CD_3)_2SO$): δ = 49.36 (CO-NCH_2), 50.76 ($NHCH_2$), 52.86 (N^+CH_2), 122.71 (CO-CH_{Ar}) 123.11 (N^+=C-CH_{Ar}), 126.39 (C_{Ar}), 126.61 (CH_{Ph}), 126.79, 127.43, 127.79, 127.85, 128.08 (all CH_{Ph}); 128.11 (CH_{Ph} + CO-CH_{Ar}), 128.69 (CH_{Ph}), 129.03 (CH_{Ph}), 131.09 (CH_{Ph}), 134.76 (C_{Ph}), 135.71 (C_{Ph}), 138.48 (C_{Ph}), 144.32 (N^+=C), 145.23 (CO-C_{Ar}), 147.10 (CO-4-C_{Ar}), 148.52 (N^+=C-4-C_{Ar}), 159.83 (CN_3), 167.23 (C=O) ppm. – MS (ESI): *m*/*z* = 655.24 $[M + H]^+$. – Anal. calcd. for $C_{36}H_{30}N_8O_5$ (654.60): C, 66.05; H, 4.62; N, 17.12; found: C, 66.00; H, 4.81; N, 16.97.

1.3.5.4. 2-Acetyl-2-benzyl-1-(1-benzyl-4-(benzylamino)-5-methyl-1*H*-1,2,4-triazol-4-ium-3-yl)hydrazin-1-ide (7d)

Method A. Under an argon atmosphere, 1,2,3-tris(benzylamino)guanidinium chloride (**4**, 1.01 g, 2.46 mmol) was suspended in acetonitrile (25 mL) and the mixture was heated at 70 °C. After addition of aqueous NaOH (5 M, 11 mL) and acetyl chloride

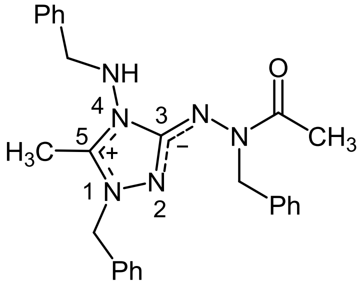

(2.0 mL, 28.0 mmol) a clear yellow-orange or red orange solution was formed, which was kept with stirring at 70 ^{o}C for 3 h.

Method B. Under an argon atmosphere, 1,2,3-tris(*N*-benzyl-acetamido)guanidine (**6d**, 150 mg, 0.30 mmol) was dissolved in acetonitrile (10 mL), aqueous NaOH (5 M, 1.7 mL) was added, and the stirred solution was heated at 70 ^{o}C for 3 h.

Workup for both methods: After cooling to room temperature, water was added and the clear solution was extracted twice with ethyl acetate (2 × 10 mL for method A, 2 × 3 mL for method B). The combined extracts were dried ($MgSO_4$) and evaporated to dryness. The residue was treated with diethyl ether in an ultrasonic bath for 30 min. The undissolved solid was isolated by suction filtration, washed with diethyl ether and dried at 40 ^{o}C/0.05 mbar to furnish a pale yellow solid. Yield, method A: 0.52 g (1.18 mmol, 48%); method B: 92 mg (0.21 mmol, 70%). M. p. 181.9–183.4 ^{o}C (heating rate 2 ^{o}C/min). Recrystallization from ethyl acetate is possible but not necessary. – IR (KBr): ν = 3444 (br, w), 3089 (w), 3030 (m), 2862 (s), 1643 (vs), 1590 (vs), 1537 (s), 1495 (w), 1454 (m), 1407 (m), 1354 (m), 1277 (w), 1245 (w), 1225 (m), 1157 (w), 1029 (w), 969 (w), 806 (w), 758 (m), 729 (m), 706 (s) cm^{-1}. – ^{1}H NMR (500.16 MHz, $(CD_3)_2SO$): δ = 1.69 (s, 3 H, N^+=C-CH_3), 1.99 (s, 3 H, $COCH_3$), 4.05 (d, ^{3}J = 2.5 Hz, 2 H, NHCH_2), 4.72 (s, 2 H, CO-NCH_2), 5.01 (s, 2 H, N^+CH_2), 6.61 (t, ^{3}J = 2.5 Hz, 1 H, NH*)* 6.96 (d, *J* = 8.5 Hz, 2 H, H_{Ph}), 7.05 (d, *J* = 10.5 Hz, 2 H, H_{Ph}), 7.15–7.24 (m, 6 H, H_{Ph}), 7.28–7.38 (m, 5 H, H_{Ph}) ppm. – ^{13}C NMR (125.76 MHz, $(CD_3)_2SO$): δ = 7.94 (N^+=C-CH_3), 20.61 (CO-CH_3), 48.62 (CO-NCH_2), 50.99 ($NHCH_2$), 51.75 (N^+CH_2); 126.27, 127.05, 127.61, 127.73, 127.86, 128.08, 128.30, 128.61, 129.72 (all CH_{Ph}); 134.87 (C_{Ph}), 136.73 (C_{Ph}), 139.26 (C_{Ph}), 145.23 (N^+=C), 158.69 (CN_3), 169.43 (C=O) ppm. – HRMS (ESI): *m*/*z* = 441.23996 $[M + H]^+$, 881.47480 $[2M + H]^+$; expected 441.23973 [$C_{26}H_{29}N_6O$], 881.47219 [$C_{52}H_{57}N_{12}O_2$]. – Anal. calcd. for $C_{26}H_{28}N_6O$ (440.54): C, 70.89; H, 6.41; N, 19.08; found: C, 71.13; H, 6.41; N, 19.14.

1.3.5.4. 2-Acetyl-2-(4-fluorobenzyl)-1-(1-(4-fluorobenzyl)-4-(4-fluorobenzylamino)-5-methyl-4*H*-1,2,4-triazol-1-ium-3-yl)hydrazin-1-ide (7e)

The compound was prepared as described for **7d** (section 1.3.5.3, method A) from 1,2,3-tris(4-fluorobenzylamino)guanidinium chloride (**5**, 2.00 g, 4.30 mmol) and acetyl chloride (2.6 mL, 16.8 mmol) in the presence of aqueous NaOH (5 M, 15 mL). Yield: 1.60 g (3.23 mmol, 75%) of a colorless powdery solid; m. p. 165.2–167.9 °C (heating rate 2 °C/min). – ^{1}H NMR (500.16 MHz, $(CD_3)_2SO$): δ = 1.77 (s, 3 H, N^+=C-CH_3), 1.98 (s, 3 H, $COCH_3$), 4.06 (broadened s, 2 H, NHCH_2), 4.71 (s, 2 H, CO-NCH_2), 5.01 (s, 2 H, N^+CH_2), 6.73 (broadened signal, 1 H, NH), 6.96–7.02 (m, 4 H, H_{Ar}), 7.03–7.10 (m, 2 H, H_{Ar}), 7.11–7.17 (m, 2 H, H_{Ar}), 7.18–7.26 (m, 2 H, H_{Ar}), 7.30–7.36 (m, 2 H, H_{Ar}) ppm. – ^{13}C NMR (125.76 MHz, $(CD_3)_2SO$): δ = 8.01 (N^+=C-CH_3), 20.65 (CH_3CO), 47.98 (CO-NCH_2), 50.04 ($NHCH_2$), 51.00 (N^+CH_2), 114.36 (d, $^2J_{C,F}$ = 21.1 Hz, *m*-CH_{Ar}), 115.01 (d, $^2J_{C,F}$ = 21.3 Hz, *m*-CH_{Ar}), 115.34 (d, $^2J_{C,F}$ = 21.6 Hz, *m*-CH_{Ar}), 129.42 (d, $^3J_{C,F}$ = 8.3 Hz, o-CH_{Ar}), 129.86 (d, $^3J_{C,F}$ = 7.9 Hz, o-CH_{Ar}), 131.10 (d, $^4J_{C,F}$ = 3.0 Hz, *i*-C_{Ar}), 131.58 (d, $^3J_{C,F}$ = 8.2 Hz, o-CH_{Ar}), 132.91 (d, $^4J_{C,F}$ = 2.9 Hz, *i*-C_{Ar}), 135.45 (d, $^4J_{C,F}$ = 2.9 Hz, *i*-C_{Ar}), 144.96 (N^+=C), 158.84 (CN_3), 160.97 (d, $^1J_{C,F}$ = 241.4 Hz, F-C_{Ar}), 161.61 (d, $^1J_{C,F}$ = 244.0 Hz, F-C_{Ar}), 161.76 (d $^1J_{C,F}$ = 244.1 Hz, F-C_{Ar}), 169.34 (C=O) ppm. – ^{19}F NMR ($CDCl_3$): δ = -116.60, -115.87, -114.86 ppm. – IR (KBr): ν = 3426 (br, w), 3140 (w), 3042 (w), 2923 (w), 1615 (s), 1510 (s), 1450 (m), 1411 (m), 1224 (s), 1157 (w), 830 (m) cm^{-1}. – Anal. calcd. for $C_{26}H_{25}F_3N_6O$ (494.52): C 63.15, H 5.10, N 16.99; calcd. for M × 0.75 H_2O: C 61.47, H 5.26, N 16.31; found: C 61.58, H 5.20, N 16.42.

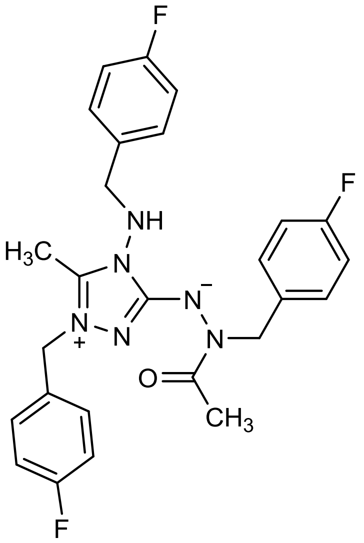

*1.3.6. Protonation and methylation of salts **7b,c***

Salt **7b** or **7c** (300 mg) was dissolved in hot methanol (5 mL). Dilute hydrochloric acid (2 M) was added till complete decoloration of the solution. Water was added and the solution was extracted with ethyl acetate. The combined organic layers were dried ($MgSO_4$) and the solvent was removed. The solid residue was dissolved in warm ethanol and the product was precipitated by addition of pentane, isolated and dried (50 °C/0.05 mbar).

1.3.6.1. 2-Benzyl-5-(*N*-benzyl-(3,4,5-trimethoxyphenyl)hydrazido)-4-(benzylamino)-3-(3,4,5-trimethoxyphenyl)-4,5-dihydro-1*H*-1,2,4-triazol-2-ium chloride (8b)

With betaine **7b** (300 mg, 0.40 mmol) the procedure afforded a colorless solid (227 mg, 0.29 mmol, 73%), m. p. 216.5–216.9 °C. – IR (KBr): ν = 3425 (w), 3185 (w), 2940 (m), 2836 (m), 1663 (m), 1620 (s), 1585 (s), 1501 (s), 1457 (s), 1415 (s), 1378 (m), 1337 (m), 1241 (s), 1182 (w), 1126 (vs), 1003 (m), 898 (w), 850 (w), 755 (w), 701 (m) cm^{-1}. – ^{1}H NMR (500.16 MHz, $(CD_3)_2SO$, 373 K): δ = 3.65–3.66 (m, 9 H, OCH_3), 3.81–3.82 (m, 11 H, OCH_3 and N^+CH_2), 4.97 (s, 2 H, CO-NCH_2), 5.26 (s, 2 H, NHCH_2), 6.49 (s, 2 H, N^+=C-CH_{Ar}), 6.56 (d, *J* = 6.6 Hz, 2 H, CH_{Ph}), 6.98 (t, *J* = 7.0 Hz, 2 H, CH_{Ph}), 7.02 (s, 2 H, CO-CH_{Ar}), 7.10–7.11 (d, *J* = 5.4 Hz, 3 H, CH_{Ph}), 7.32–7.39 (m, 6 H, CH_{Ph}), 7.61 (d, *J* = 7.1 Hz, 2 H, CH_{Ph}), 7.82 (s, 1 H, CH_2NH), 11.94 (s, 1 H, $NH_{hydrazide}$) ppm. – ^{13}C NMR (125.76 MHz, $(CD_3)_2SO$, 298 K): δ = 52.29 (broad, CH_2), 52.85 (CH_2), 53.89 (CH_2), 55.86 (OCH_3), 56.11 (OCH_3), 59.85 (OCH_3), 60.18 (OCH_3), 105.17 (CO-CH_{Ar}), 107.40 (N^+=C-CH_{Ar}), 112.92 (C=N^+); 126.68, 127.44, 127.71, 127.99, 128.32 (2 C), 128.39, 128.76, 129.05 (all CH_{Ph}); 129.53 (CO-C_{Ar}), 134.21 (C_{Ph}), 134.44 (C_{Ph}), 136.40 (C_{Ph}), 139.37 (N^+=C-3-C_{Ar}OMe), 140.79, 149.70, 152.54, 152.59 (CO-4-C_{Ar}OMe), 152.64 (N^+=C-4-C_{Ar}OMe), 171.41 (C=O) ppm. – MS (ESI): *m/z* = 745.33 $[M - Cl]^+$. – Anal. calcd. for $C_{42}H_{45}ClN_6O_7$ (781.30): C, 64.57; H, 5.81; N, 10.76; found: C, 64.50; H, 5.90; N, 10.73.

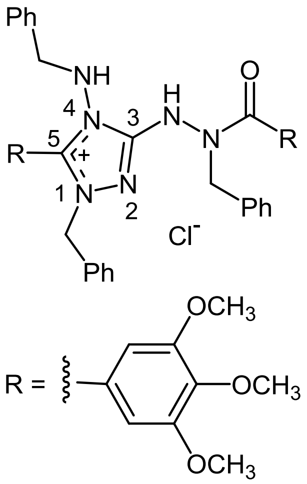

1.3.6.2. 2-Benzyl-5-(*N*-benzyl-(4-nitrophenyl)hydrazido)-4-(benzylamino)-3-(4-nitrophenyl)-4,5-dihydro-1*H*-1,2,4-triazol-2-ium chloride (8c)

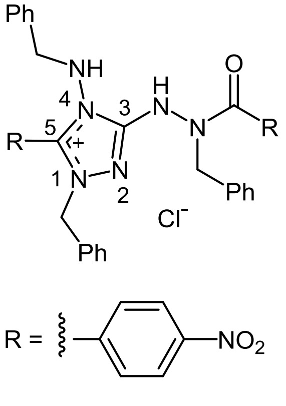

With betaine **7c** (300 mg, 0.46 mmol) the procedure afforded a yellow solid (182 mg, 0.26 mmol, 56%), m. p. 180.2–180.9 °C. – IR (KBr): ν = 3033 (br), 1675 (s), 1622 (s), 1527 (s), 1452 (m), 1411 (w), 1350 (s), 1318 (m), 1180 (w), 1107 (w), 1080 (w), 1045 (w), 985 (w), 857 (s), 750 (m), 700 (s), 626 (w) cm^{-1}. – ^{1}H NMR (500.16 MHz, $(CD_3)_2SO$, 373 K): δ = 3.73 (d, ^{3}J = 3.6 Hz, 2 H, NHCH_2), 4.96 (s, 2 H, CO-NCH_2), 5.27 (s, 2 H,

N^+CH_2). 6.52 (d, *J* = 7.3 Hz, 2 H, H_{Ph}), 6.99 (t, *J* = 7.7 Hz, 2 H, H_{Ph}), 7.12–7.14 (m, 3 H, H_{Ph}), 7.33–7.43 (m, 6 H, H_{Ph}), 7.50–7.55 (m, 3 H, CH_2NH and H_{Ph}), 7.58/8.27 (AA'BB' system, *J* = 8.8 Hz, 4 H, CO-C_6H_4), 7.82/8.23 (AA'BB system, *J* = 8.8 Hz, 4 H, N^+=C-C_6H_4), 11.00–12.00 (very broad, 1 H, $NH_{hydrazide}$) ppm. – ^{13}C NMR (125.76 MHz, $(CD_3)_2SO$, 298 K): δ = 52.74 (CH_2), 54.21 (CH_2), 123.44 (CH_{Ar}), 123.67 (CH_{Ar}), 124.12; 127.67, 128.01, 128.22, 128.44, 128.56, 128.69, 128.81, 131.97 (all CH_{Ar}); 133.08 (C_{Ph}), 134.30 (C_{Ph}), 135.73 (C_{Ph}), 140.79 (C_{Ar}), 148.35 (C_{Ar}), 148.47 (C_{Ar}), 149.64 (C_{Ar}), 152.76 (C=N) ppm. Several CH_{Ph} signals coincide and the signals for $NHCH_2$ and C=O were not visible. – MS (ESI): *m*/*z* = 655.24 [M – Cl]$^+$. – Anal. calcd. for $C_{36}H_{31}ClN_8O_5$ (564.69): C, 62.56; H, 4.52; N, 16.21; calcd. for $C_{36}H_{31}ClN_8O_5 \times 1.33\ H_2O$: C, 60.46; H, 4.74; N, 15.67; found: C, 60.50; H, 4.63; N, 15.60.

1.3.6.3. 1-Benzyl-3-(*N*-benzyl-*N*'-methyl-(3,4,5-trimethoxyphenyl)-hydrazido)-4-(benzylamino)-5-(3,4,5-trimethoxyphenyl)-4*H*-1,2,4-triazol-1-ium trifluoromethanesulfonate (9b)

Betaine **7b** (360 mg, 0.48 mmol) was dissolved in chloroform (10 mL) under an argon atmosphere and heated to reflux. A solution of methyl trifluoromethanesulfonate (84 µL, 0.76 mmol) in dry chloroform (10 mL) was added slowly. The solution was stirred for additional 1.5 hours at 70 ^{o}C, whereby it became colorless. After addition of water the emulsion was extracted with chloroform. The organic layer was dried over magnesium sulfate and the solvent was removed. The solid residue was triturated twice with pentane in an ultrasonic bath, then the solid was filtered off, briefly heated in ethyl acetate (2 mL) and after cooling was filtered off again. After drying *in vacuo* a colorless solid was obtained, which assumed an orange color at the surface when exposed to air (318 mg, 0.35 mmol, 72%), m. p. 168.9–170.0 °C. – IR (KBr): ν = 3504 (br), 3284 (w), 3065 (w), 2941 (w), 1671 (s), 1586 (s), 1504 (s), 1456 (s), 1419 (s), 1392 (s), 1349 (s), 1326 (m), 1281 (s), 1247 (s), 1159 (s), 1223 (s), 1029 (s), 999 (m), 895 (w), 839 (m), 738 (m), 700 (m), 637 (s) cm^{-1}. – ^{1}H NMR (500.16 MHz, $(CD_3)_2SO$, 373 K): δ = 3.52 (s, 3 H, NCH_3), 3.69 (s, 3 H, OMe), 3.73 (s, 6 H, OMe), 3.75–3.77 (broad, 2 H, N^+CH_2), 3.77 (s, 6 H, OMe), 3.85 (s, 3 H, OMe), 4.87 (s, 2 H, CO-NCH_2), 5.37 (s, 2

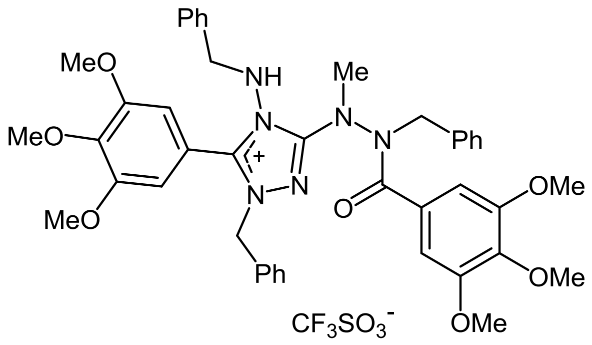

H, NHCH_2), 6.75 (d, J = 7.3 Hz, 2 H, H_{Ph}), 6.80 (s, 2 H, H_{Ar}), 6.83 (s, 2 H, H_{Ar}), 7.07 (t, J = 5.1 Hz, 1 H, H_{Ph}), 7.14 (t, J = 7.6 Hz, 2 H, H_{Ph}), 7.02–7.22 (m, 3 H, NH and H_{Ph}), 7.34–7.44 (m, 8 H, H_{Ph}) ppm. At 298 K, several signals exhibit coalescence phenomena. – ^{13}C NMR (125.76 MHz, $(CD_3)_2SO$, 298 K): δ = 53.20 (CH_2), 54.32 (CH_2), 56.01 (CH_3), 56.06 (CH_3), 56.10 (CH_3), 59.92 (CH_3), 60.27 (CH_3), 104.56 (CH_{Ar}), 107.44 (CH_{Ar}), 112.93 (weak signal in coalescence, CF_3); 127.57, 127.71, 128.09, 128.33, 128.62, 128.83, 128.90 (all CH_{Ph}); 133.51, 139.42, 141.13, 151.01, 152.76, 153.16, 153.66 (all quaternary C) ppm. – ^{19}F NMR ($(CD_3)_2SO$, 298 K): δ = -77.73 ppm. – MS (ESI): m/z = 761.35 $[M - CF_3SO_3]^+$, 1667.65 $[2M - CF_3SO_3^-]^+$. – Anal. calcd. for $C_{44}H_{47}F_3N_6O_{10}S$ (908.94): C, 58.14; H, 5.21; N, 9.25; calcd. for $C_{44}H_{47}F_3N_6O_{10}S \times 1.34\ H_2O$: C, 56.62; H, 5.38; N, 9.00; found: C, 56.66; H, 5.34; N, 8.99.

*1.3.7. Hydrogenation of betaines **7***

1.3.7.1. *N*-Benzyl-*N'*-(4-(benzylamino)-5-phenyl-4*H*-1,2,4-triazol-3-yl)benzohydrazide (10a)

Betaine **7a** (251 mg, 0.44 mmol) was dissolved in methanol (25 mL). After addition of Pd/C (10%, 26 mg) the solution was stirred for 24 h at room temperature under an

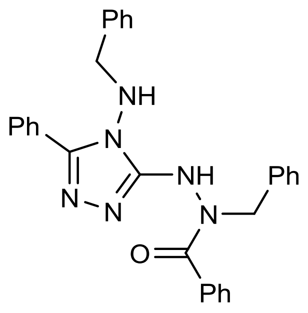

atmosphere of H_2 (50 mbar overpressure). The catalyst was filtered off using a syringe filter. The filtered solution was evaporated to dryness and the residue was suspended in diisopropyl ether (3 mL) and exposed to ultrasonication for 10 min. The solid was filtered off and dried (40 °C, 0.05 mbar), affording a colorless solid (172 mg, 0.36 mmol, 81%), m. p. 215.3–216.4 °C. – ^{1}H NMR (500.16 MHz, $(CD_3)_2SO$, 356 K): δ = 3.55 (d, ^3J = 4.5 Hz, 2 H, CH_2NH), 4.95 (s, 2 H, CO-NCH_2), 6.31 (t, ^3J = 5.0 Hz, 1 H, NHCH_2Ph), 6.85–6.92 (m, 2 H, H_{Ph}), 7.10–7.18 (m, 3 H, H_{Ph}), 7.30–7.35 (m, 1 H, H_{Ph}), 7.40–7.48 (m, 10 H, H_{Ph}), 7.60–7.65 (m, 2 H, H_{Ph}), 7.82–7.88 (m, 2 H, H_{Ph}), 8.61 (s, 1 H, NHNCH_2) ppm. – ^{13}C NMR (125.76 MHz, $(CD_3)_2SO$, 298 K): δ = 50.58 (CO-NCH_2), 53.38 (NHCH_2Ph); 126.98, 127.23, 127.32, 127.55, 127.83, 128.03, 128.13, 128.38, 128.48, 128.96, 129.25, 129.87 (all CH_{Ph}); 135.31, 135.66, 136.83 (all C_{Ph}); 149.79 (Ph-$C_{triazole}$=N *), 153.35 (CN_3 *), 172.63 (C=O) ppm; * = tentative assignment, based on HMBC spectra and ^nJ(C,H) coupling. – HRMS (ESI): m/z = 475.2241 (calcd.

475.2241 for $C_{29}H_{27}N_6O$, $[M + H]^+$), 949.4431 (calcd. 949.4409 for $C_{58}H_{53}N_{12}O_2$, $[2M + H]^+$). – IR (KBr): ν = 3443 (br, m), 3223 (br, m), 3059 (w), 3030 (w), 2922 (w), 1663 (s), 1575 (s), 1481 (m), 1449 (m), 1410 (m), 1280 (w), 1258 (w), 1076 (w), 970 (w), 751 (m), 694 (s) cm^{-1}. – Anal. calcd. for $C_{29}H_{26}N_6O$ (474.57): C 73.40, H 5.52, N 17.71; found: C 73.40, H 5.52, N 17.63.

1.3.7.2. *N*-Benzyl-*N'*-(4-(benzylamino)-5-(3,4,5-trimethoxyphenyl)-4*H*-1,2,4-triazol-3-yl)-3,4,5-trimethoxybenzohydrazide (10b)

Betaine **7b** (500 mg, 0.67 mmol) was dissolved in methanol (20 mL). After addition of Pd/C (10%, 50 mg) the solution was stirred for 22 h at room temperature under an atmosphere of H_2 (50 mbar overpressure). The catalyst was filtered off using a syringe filter. The filtered solution was evaporated to dryness and the residue was dissolved in as little ethanol as possible. By addition of diethyl ether a solid could be precipitated which was filtered off and dried (50 °C, 0.05 mbar) affording a colorless solid (400 mg, 0.61 mmol, 91%), m. p. 164.6–164.9 °C. – IR (KBr): ν = 3504 (br, m, OH of H_2O), 3218 (m), 2943 (m), 1666 (m), 1586 (m), 1491 (m), 1459 (m), 1414 (m), 1357 (w), 1323 (m), 1239 (m), 1184 (w), 1128 (s), 1005 (m), 845 (m), 757 (m), 699 (m) cm^{-1}. – ^1H NMR (500.16 MHz, $(CD_3)_2SO$, 373 K): δ = 3.58 (d, ^3J = 5.3 Hz, 2 H, NHCH_2), 3.63 (s, 3 H, OCH_3), 3.72 (s, 6 H, OCH_3), 3.78 (s, 3 H, OCH_3), 3.79 (s, 6 H, OCH_3), 4.93 (s, 2 H, CO-NCH_2), 6.35 (t, ^3J = 5.3 Hz, 1 H, N*H*CH_2), 6.92–6.93 (m, 2 H, H_{Ph}), 6.93 (s, 2 H, H_{Ar}), 7.12–7.16 (m, 3 H, H_{Ph}), 7.16 (s, 2 H, H_{Ar}) 7.30 (t, J = 7.3 Hz, 1 H, H_{Ph}), 7.38 (t, J = 7.4 Hz, 2 H, H_{Ph}), 7.46 (d, J = 7.2 Hz, 2 H, H_{Ph}), 8.59 (s, 1 H, N*H*NCH_2) ppm. At 298 K, several signals are in coalescence. – ^{13}C NMR (125.76 MHz, $(CD_3)_2SO$, 298 K): δ = 51.05 (CO-NCH_2), 53.37 (NH*C*H_2), 55.83 (OCH_3), 56.06 (OCH_3), 59.86 (OCH_3), 60.13 (OCH_3), 104.58 (CH_{Ar}), 104.60 (CH_{Ar}), 122.66 (C_{Ar}), 127.29, 127.54, 128.00, 128.40, 128.77 (all CH_{Ph}); 130.89 (CO-C_{Ar}), 135.46 (C_{Ph}), 136.82 (C_{Ph}), 138.52 (N=C-3-C_{Ar}-OMe), 138.69 (CO-3-C_{Ar}-OMe), 149.47 (Ph-$C_{triazole}$=N *), 152.30 (CO-4-C_{Ar}-OMe) 152.87 (N=C-4-C_{Ar}-OMe), 153.60 (CN_3 *), 172.29 (CO) ppm; * = tentative assignment, based on HMBC spectra and ^nJ(C,H) coupling). Due to coalescence processes, some of the ^{13}C signals were not visible in a conventional

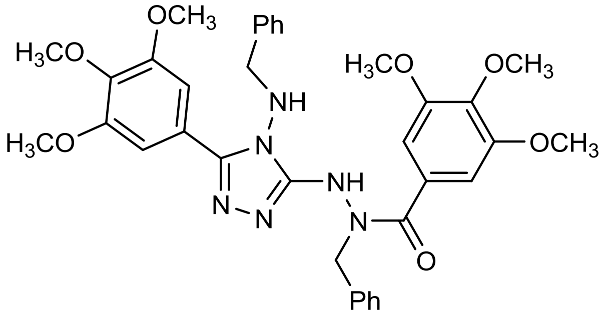

^{13}C spectrum, but could be detected when the UDEFT sequence [3] was applied. – HRMS (MALDI-TOF): *m*/*z* = 655.28725 $[M + H]^+$; calculated 655.28747 $[C_{35}H_{39}N_6O_7]^+$. – Anal. calcd. for $C_{35}H_{38}N_6O_7$ (654.72): C, 64.21; H, 5.85; N, 12.84; found: C, 64.25; H, 5.93; N, 12.76.

1.3.7.3. 4-Amino-*N'*-(5-(4-aminophenyl)-4-(benzylamino)-4*H*-1,2,4-triazol-3-yl)-*N*-benzyl-benzohydrazide (10c)

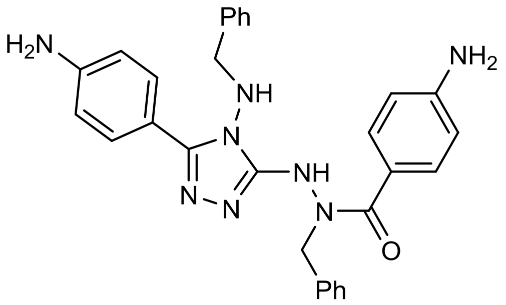

Betaine **7c** (0.40 g, 0.61 mmol) was dissolved in methanol (25 mL). After addition of Pd/C (10%, 42 mg) the solution was stirred for 48 h at room temperature under an atmosphere of H_2 (50 mbar overpressure). The catalyst was filtered off using a syringe filter. The filtered solution was evaporated to dryness and the residue was dissolved in as little methanol as possible, and this solution was gradually added to diethyl ether (10 mL). The precipitated solid was filtered off and dried (40 °C, 0.05 mbar) to furnish 0.17 g (0.34 mmol, 56%) of a colorless powdery solid, m. p. 170.0–172.2 °C. – ^{1}H NMR (500.16 MHz, $(CD_3)_2SO$, 355 K): δ = 3.77 (d, ^3J = 5.5 Hz, 2 H, CH_2NH), 4.89 (s, 2 H, NCH_2Ph), 5.00–5.40 (broad unstructured signal, 4 H, NH_2) 6.25 (broadened s, 1 H, N*H*CH_2), 6.55/6.65 (AA'BB' spin system, ^3J = 8.5 Hz, 4 H, H_{Ar}), 7.16–7.22 (m, 3 H, H_{Ar}), 7.26–7.30 (m, 1 H, H_{Ar}), 7.32–7.37 (m, 2 H, H_{Ar}), 7.38–7.42 (m, 2 H, H_{Ar}), 7.45/7.60 (AA'BB', ^3J = 8.6 Hz, 4 H, H_{Ar}), 8.25 (broadened s, 1 H, N*H*NCH_2) ppm. – ^{13}C NMR (125.76 MHz, $(CD_3)_2SO$, 298 K): δ = 51.60 (CO-NCH_2), 53.42 (CH_2NH), 112.11 (CH_{Ar}), 113.30 (CH_{Ar}), 114.47 (C_{Ar}), 120.98 (C_{Ar}); 127.01, 127.53, 127.93, 127.99, 128.11, 128.29, 128.98, 130.00 (all CH_{Ar}); 135.71 (C_{Ar}), 137.47 (C_{Ar}), 149.82 and 150.06 (C_{Ar}-$C_{triazole}$=N * and $C_{Ar}NH_2$), 151.06 ($C_{Ar}NH_2$) 152.93 (CN_3 *), 172.99 (C=O) ppm; * = tentative assignment, based on HMBC spectra and ^nJ(C,H) coupling). – MS (CI): *m*/*z* = 505 $[M + H]^+$. – IR (KBr): ν = 3449 (br, m), 3349 (br, s), 3216 (br, s), 3059 (w), 3030 (w), 2927 (w), 1607 (vs), 1573 (s), 1517 (m), 1490 (s), 1438 (m), 1389 (m), 1347 (m), 1296 (s), 1180 (s), 982 (w), 835 (s), 754 (m), 734 (m), 701 (s) cm^{-1}. – Anal. calcd. for $C_{29}H_{28}N_8O$ (504.60): calcd. C 69.03, H 5.59, N 22.21; calcd. for M × 0.50 H_2O: C 67.82, H 5.69, N 21.82; found: 67.81, H 5.59, N 21.53.

1.3.7.4. *N*-Benzyl-*N*'-(4-(benzylamino)-5-methyl-4*H*-1,2,4-triazol-3-yl]acetohydrazide (10d)

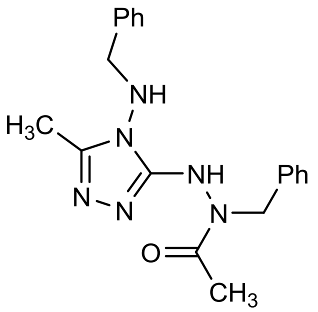

Betaine **7d** (241 mg, 0.55 mmol) was dissolved in methanol (25 mL). After addition of Pd/C (10%, 24 mg) the solution was stirred for 24 h at room temperature under an atmosphere of H_2 (50 mbar overpressure). The catalyst was filtered off using a syringe filter. The filtered solution was evaporated to dryness; the residue was suspended in diethyl ether (4 mL) and exposed to ultrasonication for 10 min. The undissolved solid was filtered off and dried (40 °C, 0.05 mbar) to furnish 150 mg (0.43 mmol, 78%) of a colorless powder, m. p. 206.1–207.6 °C. – ^{1}H NMR (500.16 MHz, $(CD_3)_2SO$, 357 K): δ = 2.07 (s, 3 H, CH_3), 2.08 (s, 3 H, CH_3), 3.96 (d, ^3J = 5.1 Hz, 2 H, CH_2NH), 4.73 (s, 2 H, CO-NCH_2), 6.33 (t, ^3J = 4.9 Hz, 1 H, N*H*CH_2), 7.22–7.31 (m, 10 H, H_{Ph}), 8.10 (s, 1 H, N*H*NCH_2) ppm. – ^{13}C NMR (125.76 MHz, $(CD_3)_2SO$, 298 K): δ = 9.79 (CH_3-$C_{triazole}$), 20.60 (CH_3CO), 49.53 (CO-NCH_2), 53.94 (CH_2NH); 127.13, 127.73, 128.13, 128.27, 128.30, 129.43 (all CH_{Ph}); 136.35 (C_{Ph}), 137.09 (C_{Ph}), 148.62 (CH_3-$C_{triazole}$=N), 151.64 (CN_3 *), 172.99 (C=O) ppm; * = tentative assignment, based on HMBC spectra and ^nJ(C,H) coupling). – HRMS (ESI): *m/z* = 351.1925 (calcd. 351.1928 for $C_{19}H_{23}N_6O$, [M + H]$^+$), 701.3771 (calcd. 701.3783 for $C_{38}H_{45}N_{12}O_2$, [2M + H]$^+$). – IR (KBr): ν = 3447 (br, w), 3176 (m), 3026 (w), 3002 (w), 2930 (w), 2858 (w), 1676 (vs), 1585 (s), 1558 (m), 1439 (m), 1393 (s), 1353 (m), 1266 (m), 1207 (m), 756 (m), 734 (m), 701 (m) cm^{-1}. – Anal. calcd. for $C_{19}H_{22}N_6O$ (350.43): calcd. C 65.12, H 6.33, N 23.98; found: C 65.26, H 6.33, N 23.92.

Table S1: Chemical shifts (δ, ppm) of the CH_2 groups of **7a–e**.

Compound	δ (CH_2-NH)	δ (CH_2-N-CO)	δ (CH_2-$N_{triazole}$)
**7a** (R = Ph)	3.64 / 50.91	4.91 / 49.16	5.00 / 52.47
**7b** (R = 3,4,5-trimethoxyphenyl)	3.74 / 51.13	4.91 / 49.66	5.06 / 52.85
**7c** (R = 4-nitrophenyl)	3.67 / 50.76	4.92 / 49.36	5.04 / 52.86
**7d** (R = Me)	4.05 / 50.99	4.72 / 48.62	5.01 / 51.75
**7e** (R = Me, Ar = 4-fluorophenyl)	4.06 / 50.04	4.71 / 47.98	5.01 / 51.00

2. X-Ray crystal structure determinations

Suitable crystals were obtained by slow diffusion of pentane vapor into an ethanolic solution of **6b**, **7a** or **9b**, and a chloroform/methanol solution of **8b**.

Data collection was performed on an Oxford Diffraction instrument (SuperNova, Dual Source, Atlas CCD). Software for structure solution and refinement: SHELXS/L-97 [4, 5] and SHELXL-2014/6 [6]; molecule plots: ORTEP-3 for Windows [7], OLEX2 [8] and Mercury, version 3.5 [9]. The hydrogen atoms were generally included in the refinement procedure in geometrically calculated positions and treated by the riding model. As an exception, the positional coordinates of the NH and OH protons were taken from a ΔF map and their positional and thermal parameters were included in the refinement procedure.

In the crystal structure of **9b** residual electron density was detected, which could not be assigned; its contribution to the structure factors was removed using the SQEEZE routine of PLATON [10].

Further details are provided in Table 1. CCDC 1055901 (**6b**), 1055902 (**7a**), 1055903 (**8b**) and 1055904 (**9b**) contain the supplementary crystallographic data for this paper. These data can be obtained free of charge from The Cambridge Crystallographic Data Centre *via* www.ccdc.cam.ac.uk/data_request/cif.

Table S2: Crystal structure data for **6b**, **7a**, **8b** and **9b**.

	**6b**	**7a**	**8b**	**9b**
Formula	$C_{52}H_{58}N_6O_{12}$ × $2C_2H_5O$	$C_{36}H_{32}N_6O$ × $0.5C_2H_5OH$	$(C_{42}H_{45}N_6O_7)^+$ Cl^-	$(C_{43}H_{47}N_6O_7)^+$ × $(CF_3SO_3)^-$ × H_2O
M_r	1049.16	587.71	781.29	926.95
Cryst. size, mm^3	0.18 × 0.12 × 0.07	0.27 × 0.20 × 0.16	0.31 × 0.18 × 0.12	0.25 × 0.14 × 0.07
Crystal system	monoclinic	triclinic	orthorhombic	triclinic
Space group	$P2_1/n$	*P*-1	$P2_12_12_1$	*P*-1
a, Å	19.3317(7)	11.1045(2)	9.6102(1)	10.4159(5)
b, Å	12.9641(4)	13.0380(4)	17.0080(2)	12.3294(6)
c, Å	21.8523(7)	23.0234(7)	25.0595(3)	19.4883(8)
α, deg	90.00	105.714(3)	90.00	85.786(4)
β, deg	99.126(3)	99.201(2)	90.00	84.483(4)
γ, deg	90.00	93.130(2)	90.00	67.120(5)
V, $Å^3$	5407.3(3)	3150.61(15)	4095.99(9)	2293.34(18)
Z	4	4	4	2
D_{calcd}, g cm^{-3}	1.289	1.239	1.267	1.342
$\mu(MoK_\alpha)$, mm^{-1}	0.767	0.615	1.290	0.148
F(000), e	2232.0	1244.0	1648.0	972.0
Radiation	CuK_α	CuK_α	CuK_α	MoK_α
Temperature, K	180(2)	180.0(1)	180.0(1)	180.0(1)
hkl range	$-23 \le h \le 20$, $-12 \le k \le 15$, $-26 \le l \le 26$	$-10 \le h \le 13$, $-15 \le k \le 16$, $-28 \le l \le 28$	$-9 \le h \le 12$, $-21 \le k \le 19$, $-29 \le l \le 31$	$-13 \le h \le 12$, $-15 \le k \le 15$, $-24 \le l \le 23$
2θ range, deg	6.66 – 145.06	7.08 – 153.4	6.28 – 152.6	5.76 – 52.74
Refl. measured	20087	31946	15245	20617
Refl. unique	10503	13049	7695	9354
R_{int}	0.0266	0.0235	0.0273	0.0304
Param. refined / restraints	712 / 3	783 / 0	519 / 0	601 / 0
$R(F)/wR(F^2)^a$ ($I \ge 2\sigma(I)$)	0.0693/0.2002	0.0447/0.1237	0.0341/0.0868	0.0692/0.2062
$R(F)/wR(F^2)^a$ (all reflexions)	0.1002/0.2279	0.0507/0.1274	0.0375/0.0901	0.0880/0.2208
GoF $(F^2)^a$	1.054	1.075	1.026	1.051
Flack parameter			-0.018(11)	
$\Delta\rho_{fin}$ (max/min), e $Å^{-3}$	0.72/-0.41	0.37/-0.35	0.20/-0.21	0.59/-0.57
CCDC	1055901	1055902	1055903	1055904

[a] $R(F) = \Sigma||F_o| - |F_c| / \Sigma|F_o|$; $wR(F^2) = [\Sigma(w(F_o^2 - F_c^2)^2) / \Sigma w(F_o^2)^2]^{1/2}$; $GoF = [\Sigma w(F_o^2 - F_c^2)^2 / (n_{obs} - n_{param})]^{1/2}$.

References

1. Weiss, S.; Krommer, H. (SKW Trostberg AG), DE3341645 (A1), 1985; *Chem. Abstr.* 1986, *104*, 206730.
2. Bucher, N.; Szabo, J.; Oppel, I. M.; Maas, G. *Z. Naturforsch.* 2012, *67b*, 631–642.
3. Piotto, M.; Bourdonneau, M.; Elbayed, K.; Wieruszeski, J.-M., Lippens, G. *Magn. Reson. Chem.* 2006, *44*, 943–947.
4. Sheldrick, G. M., SHELXS/L-97, Program for the Solution and Refinement of Crystal Structures from Diffraction Data, University of Göttingen, Göttingen (Germany), 1993–2014.
5. Sheldrick, G. M. *Acta Crystallogr.* 2008, *A64*, 112–122.
6. Sheldrick, G. M. *Acta Crystallogr*. 2015, *C71*, 3–8.
7. (a) Farrugia, L. J. *J. Appl. Cryst.* 2012, *45*, 849–854. (b) Farrugia, L. J., ORTEP, Windows version, University of Glasgow, Glasgow (Scotland), 1997–2008.
8. Dolomanov, O. V.; Bourhis, L. J.; Gildea, R. J.; Howard, J. A. K.; Puschmann, H. J*. Appl. Crystallogr.* 2009, *42*, 339–341.
9. Mercury, version 3.5, Cambridge Crystallographic Data Centre, 2001–2012.
10. (a) Spek, A. L. *Acta Crystallogr., Sect A* 1990, *46*, C34. (b) Spek, A. L., PLATON, A Multipurpose Crystallographic Tool, Utrecht University, Utrecht, The Netherlands, 1998.
